# Akuammiline alkaloid derivatives: divergent synthesis and effect on the proliferation of rheumatoid arthritis fibroblast-like synoviocytes

**DOI:** 10.3389/fchem.2023.1179948

**Published:** 2023-04-28

**Authors:** Xinye Bao, Jian Wei, Cheng Tao, Muhammad Adnan Bashir, Hai-Jun Zhang, Bian Bao, Jian Chen, Hongbin Zhai

**Affiliations:** ^1^ School of Chemical Biology and Biotechnology, Shenzhen Graduate School of Peking University, Shenzhen, China; ^2^ Guangzhou Quality Supervision and Testing Institute, Guangzhou, China; ^3^ Guangdong Provincial Key Laboratory of Research and Development of Natural Drugs, and School of Pharmacy, Guangdong Medical University, Dongguan, China; ^4^ Department of Rheumatism and Immunology, Peking University Shenzhen Hospital, Shenzhen, China

**Keywords:** alkaloid derivates, rheumatoid arthritis, fibroblast-like synoviocytes, structure activity relationships, anti-inflammatory

## Abstract

During the past decades, rheumatoid arthritis had become a serious problem, torturing millions of patients because of unclear pathogenesis and no ideal therapies. Natural products remain an important source of medicines to treat various major diseases such as rheumatoid arthritis (RA) given their excellent biocompatibility and structural diversity. Herein, we have developed a versatile synthetic method for constructing various skeletons of akuammiline alkaloid analogs based on our previous research on the total synthesis of the related indole alkaloids. We have also evaluated the effect of these analogs on the proliferation of RA fibroblast-like synoviocytes (FLSs) *in vitro* and analyzed the corresponding structure-activity relationship (SAR). Among these analogs, compounds **9** and **17c** have demonstrated a promising inhibitory effect on the proliferation of RA-FLSs, with IC_50_ values of 3.22 ± 0.29 μM and 3.21 ± 0.31 μM, respectively. Our findings provide a solid foundation for future pharmacological studies on akuammiline alkaloid derivatives and inspiration for the development of anti-RA small molecule drugs derived from natural products.

## 1 Introduction

It is widely acknowledged that human beings are affected by a range of chronic illnesses, including the potentially debilitating rheumatoid arthritis (RA). If patients with RA are not treated in a timely manner, it could lead to irreversible joint damage and further deterioration over time. During the past decades, chemotherapy that utilizes chemical drugs for RA treatment has held a dominant position in RA therapy. However, emergent drug resistance and undesirable side effects still exist and impede the treatment of RA. (Smolen et al., 2018). Hence, the development of small molecule anti-RA drugs is still highly desirable.

Natural products, due to their good biocompatibility and structural diversity, have acted as an important source of medicines in treating major diseases such as RA. (Yu et al., 2022). Many natural products, such as flavonoids, polyphenols, terpenoids, and alkaloids possessing anti-inflammatory properties, have been used clinically for this purpose. (Ma and Jiang, 2016; Wang et al., 2021; Guo et al., 2022). Multiple component extracts from plants (including herbs), such as *Melodinus henryi* extracts, Betelvine (*Piper betle* L.) extracts, and Fuzi lipid-soluble alkaloids, have been reported for RA treatment. (Li et al., 2019; Li et al., 2020; Murugesan et al., 2020; Yang et al., 2020). Scientists continue to research natural products, aiming to find more effective and viable treatments for chronic diseases. For instance, Shen *et al.* (2020) reported the mechanism of berberine treatment, which involved the inhibition of cell proliferation of fibroblast-like synoviocytes (FLSs), the effector cells of synovial hyperplasia of RA, via immunomodulatory effects. Moreover, Zhang and coworkers found that an indole alkaloid, Tabersonine (Tab), decreased the expression of inflammatory factors and reduced the proliferation of FLSs, in a manner similar to berberine. (Zhang et al., 2022a). However, akuammiline alkaloid-based small molecules for RA treatment have been investigated less. The structurally complex akuammiline alkaloids have been challenging to synthesize and thus create an abundant synthetic supply for biological investigations, which has hampered their potential to be developed into innovative drugs. 

Since the unique indole heterocycle moiety of akuammiline alkaloids present in many FDA-approved marketed anti-inflammatory medicines generally correlates to the biological activities of the compounds, (Estevao et al., 2010; Guerra et al., 2011; Kumari and Singh, 2019; Sharma et al., 2021; Grau et al., 2023), it is reasonable to simplify the structure of the original akuammiline alkaloids by preserving the key indole heterocycle moiety for novel small molecule anti-inflammatory drug development.

**SCHEME 1:**
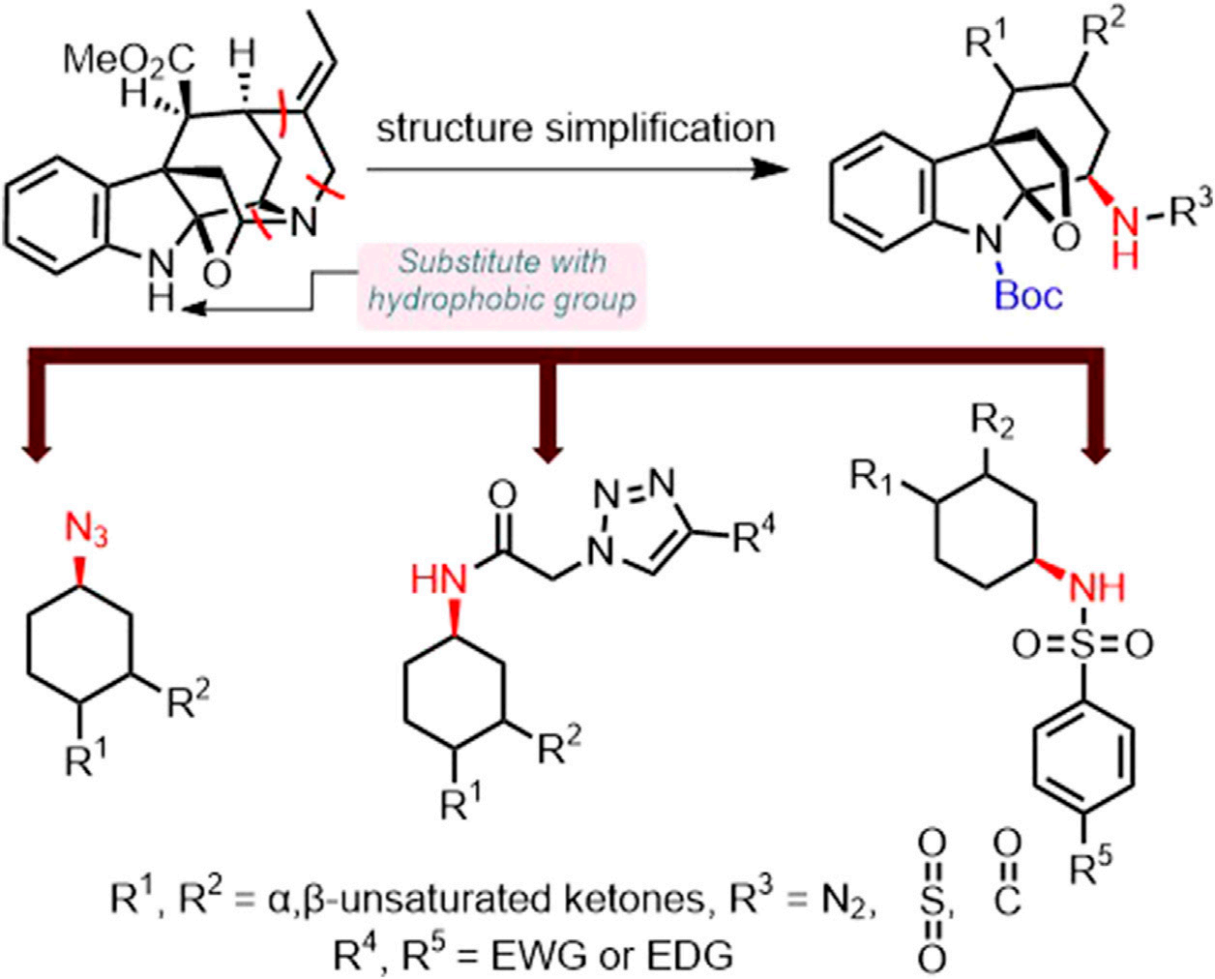
The primary design of akuammiline alkaloid derivatives.

Azido is a common functional group found in many small molecule drugs, including the classic antiviral drug zidovudine (AZT). Also, the presence of the azido moiety was found to possess a bone-targeting effect. (Zhang et al., 2021a). Moreover, triazoles and sulfonamides are important fragments present in the structure of marketed small molecule drugs with a wide range of bioactivities such as antibacterial, analgesic, anti-inflammatory, anti-tumor, and antiviral activities, and thus possess excellent pharmacological applications. (Haider et al., 2013; Abdellatif et al., 2016; Bonandi et al., 2017; Akgul et al., 2018; Pandit et al., 2018; Bozorov et al., 2019; Zhang, 2019; Shaker et al., 2020; Zhang et al., 2021b; Dai et al., 2022; Du et al., 2023). Therefore, we hypothesized that the introduction of azido and sulfonyl groups to the simplified structure of akuammiline alkaloid picrinine might result in improved and complementary anti-RA effects. According to the above hypothesis, we designed a series of akuammiline alkaloid derivatives (Scheme 1).

Based on our prior research on the total synthesis of the akuammiline alkaloids, (Wang et al., 2017; Zou et al., 2021), a flexible synthetic process was established for creating the analogs of the akuammiline alkaloids with various skeletons and the effect of these compounds on the proliferation of RA-FLSs *in vitro* was investigated.

## 2 Results and discussion

### 2.1 Chemical synthesis

**SCHEME 2:**
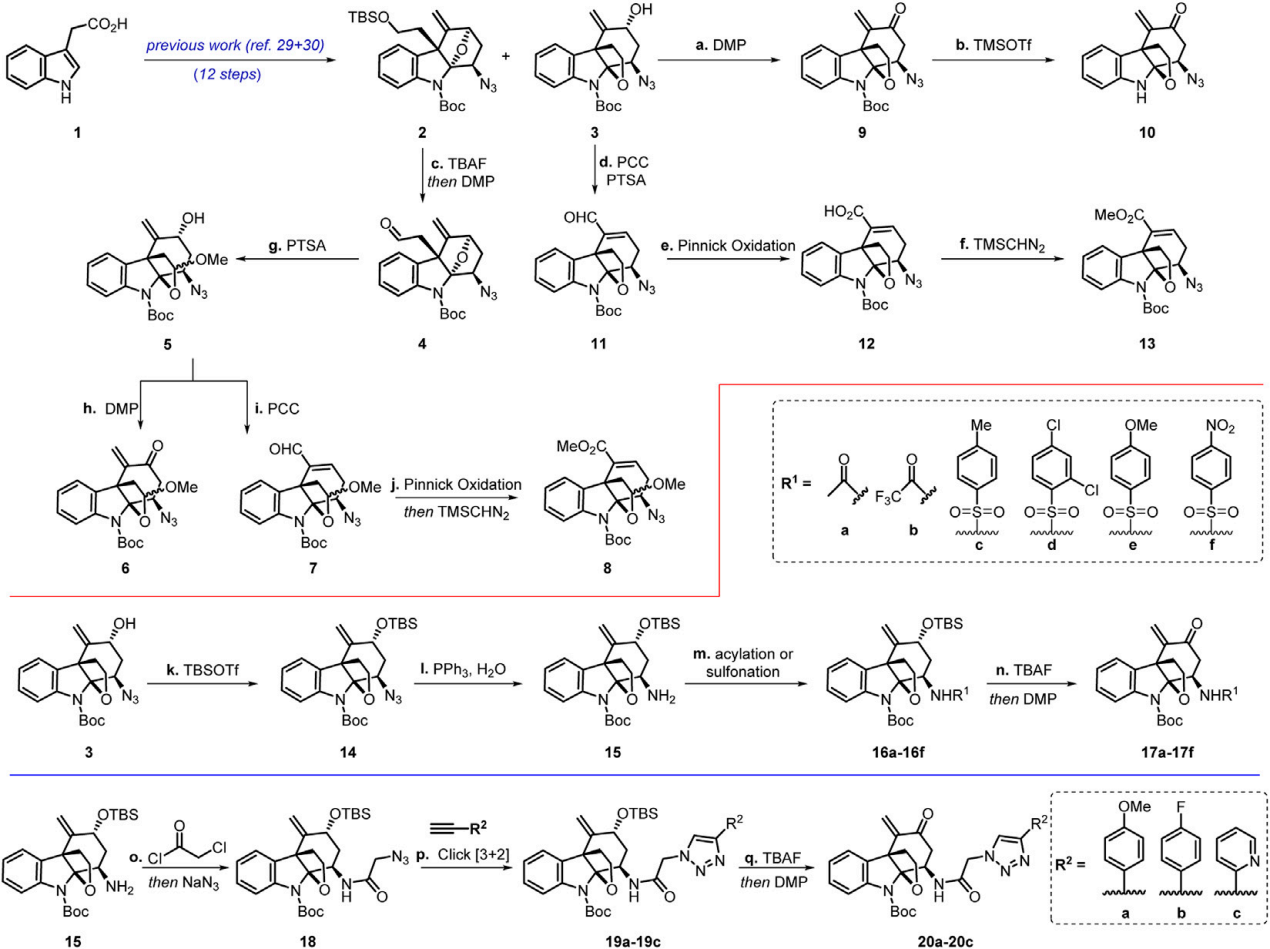
Chemical synthesis of the Akuammiline alkaloid derivatives. Reagents and conditions: **(A)** DMP (1.5 equiv), NaHCO_3_ (2 equiv), DCM, 0°C, 85%; **(B)** TMSOTf (3 equiv), DCM, 0°C, 40%; **(C)** TBAF (3 equiv), THF, 50°C; then DMP (1.5 equiv), NaHCO_3_ (2 equiv), DCM, 0°C, 82%; **(D)** PTSA (0.1 equiv), MeOH, reflux; then remove MeOH, PCC (2 equiv), PTSA (1 equiv), DCM, reflux, 63%; **(E)** NaClO_2_ (10 equiv), NaH_2_PO_4_·2H_2_O (10 equiv), 2-methyl-2-butene (10 equiv), ^t^BuOH/H_2_O = (1:1), 0 °C to rt, 89%; **(F)** TMSCHN_2_ (2 equiv), PhMeOH/MeOH = (10:1), 0 °C to rt, 78%; **(G)** PTSA (0.1 equiv), MeOH, reflux, 88%; **(H)** DMP (1.5 equiv), NaHCO_3_ (2 equiv), DCM, 0°C, 86%; **(I)** PCC (2 equiv), PTSA (1 equiv), DCM, reflux, 75%; **(J)** NaClO_2_ (10 equiv), NaH_2_PO_4_.2H_2_O (10 equiv), 2-methyl-2-butene (10 equiv), ^t^BuOH/H_2_O = (1:1), 0 °C to rt; then TMSCHN_2_ (2 equiv), PhMeOH/MeOH = (10:1), 0 °C to rt, 59%; **(K)** TBSOTf (1.2 equiv), 2,6-lutidine (2 equiv), DCM, 0°C, 80%; **(L)** PPh_3_ (3 equiv), H_2_O, THF, 50°C, 66%; (m) (*para*-substituted) benzenesulfonyl chlorides (1.1 equiv), Et_3_N (2 equiv), DCM, 0°C, 84–93%; (n) TBAF (5 equiv), THF, 60°C; then DMP (1.5 equiv), NaHCO_3_ (2 equiv), DCM, 0°C, 62–78%; (o) chloroacetyl chloride (1.1 equiv), Et_3_N (2 equiv), DCM, 0°C; then NaN_3_ (3 equiv), DMF/H_2_O = (3:1), rt, 64%; (p) three (para-substituted) benzene acetylene (1.1 equiv), CuSO_4_·H_2_O (0.2 equiv), sodium ascorbate (0.2 equiv), ^t^BuOH/H_2_O = (1:1), rt, 65–72%; (q) TBAF (5 equiv), THF, 60°C; then DMP (1.5 equiv), NaHCO_3_ (2 equiv), DCM, 0°C, 60–71%.

Our work commenced with the synthesis of two known intermediates **2** and **3** (Scheme 2). (Wang et al., 2017; Zou et al., 2021) According to our previous work, we prepared the intermediates with two different polycyclic skeletons through an eight-step protocol featuring a gold(I)-catalyzed cascade cyclization for the construction of the α,β-acetylenic ketone moiety as well as a stereoselective introduction of azido groups via the azidoalkoxylation reaction. (Wang et al., 2017; Zou et al., 2021).

DCM = dichloromethane, DMF = *N*, *N*-Dimethylformamide, DMP = Dess−Martin periodinane, THF = tetrahydrofuran, TBAF = tetra-n-butylammonium fluoride, PCC = pyridinium chlorochromate, PTSA = *p*-toluenesulfonic acid, TMSOTf = trimethylsilyl triflate, TBSOTf = *tert*-butyldimethylsilyl triflate.

Since both **2** and **3** contain the common azido moiety, we undertook structural derivatization by introducing other functional groups to these compounds. From compound **2,** we explored the ring opening of the tetrahydrofuran unit to form the cyclic acetal moiety. Desilylation of compound **2** with TBAF followed by oxidation with DMP afforded aldehyde **4**. Treatment of **4** with PTSA affected the ring opening reaction of the tetrahydrofuran moiety and the cyclization to afford acetals **5** as a mixture of two inseparable diastereomers in a 63% combined yield (d.r. = 2:1). Compound **6** (also containing two inseparable diastereomers) was obtained from **5** by oxidation with DMP. On the contrary, compound **7** was delivered upon treatment of **5** with PCC, presumably through an oxidative transposition of the secondary allyl alcohol. Aldehyde **7** was further oxidized with NaClO_2_ to furnish a carboxylic acid, the esterification of which with TMSCHN_2_ generated methyl esters **8**. Meanwhile, intermediate **3** was oxidized with DMP to give enone **9**, which was transformed into compound **10** upon Boc deprotection with TMSOTf. Furthermore, starting from allylic alcohol **3**, α,β-unsaturated aldehyde **11**, acid **12**, and ester **13** were synthesized in a fashion analogous to that for the preparation of compounds **7** and **8**.

In an endeavor to introduce a triazole or a sulfonyl group, the hydroxyl in intermediate **3** was protected as a TBS ether to lead to **14** in a 66% yield. Reduction of the azido group fraction of **14** under the Staudinger conditions produced the corresponding primary amine **15**. Compound **15** was acylated or sulfonylated to afford a series of amides or sulfonamide intermediates (**16a-16f**). For sulfonamides, there is an electron-donating or electron-withdrawing group at the *para* position. Subsequently, desilylation of **16a-16f** with TBAF followed by oxidation with DMP afforded compounds **17a-17f**.

The azido group in **14** failed to undergo the [3 + 2] cycloaddition reaction, presumably due to the steric hindrance. Upon treatment with chloroacetyl chloride followed by an S_N_2 reaction with NaN_3_, amine **15** was converted into azide **18** containing a more accessible azido group compared to that in **14**. Click reaction of **18** with three different terminal alkynes formed 1,2,3-triazoles **19a-19c** in 65–72% yields. Desilylation of **19a-19c** with TBAF followed by oxidation with DMP furnished enones **20a-20c** in 60–71% yields.

As such, three types of desirable akuammiline alkaloid derivatives were obtained, including azides **2**-**14**, amides/sulfonamides **16a-16f** and **17a-17f**, and triazoles **19a-19c** and **20a-20c.** Biological studies of these compounds were subsequently conducted.

### 2.2 Effect of compounds on the proliferation of RA-FLSs

Considering that FLSs are effector cells of synovial hyperplasia of RA, the effect of the compounds on the viability of RA-FLSs was assessed after stimulation for 24 h in the current study. As shown in Table 1, the 24 h-half maximal inhibition concentrations (IC_50_) of **6**, **9**, **17a**, **17c**, **17d**, and **17f** on MH7A cells were less than 10 μM, among which **9** and **17c** exhibited the optimal inhibitory effect on the proliferation of MH7A cells with an IC_50_ of 3.22 ± 0.29 μM and 3.21 ± 0.31 μM, respectively. These results suggested that **9** and **17c** may have the potential to be applied to treating RA.

**TABLE 1:** 24h**-**IC_**50**_ **of compounds on MH7A cells**.

Entry	Compound	24 h-IC_50_ (μM)*^a^*
1	3	>10
2	4	>10
3	5	>10
4	6	5.67 ± 0.37
5	7	>10
6	8	>10
7	9	3.22 ± 0.29
8	10	>10
9	13	>10
10	17a	7.96 ± 0.46
11	17b	>10
12	17c	3.21 ± 0.31
13	17d	9.14 ± 0.55
14	17e	>10
15	17f	8.34 ± 0.53
16	20a	>10
17	20b	>10
18	20c	>10
19	(−)-picrinine	>10
20	Methotrexate	>10

^a^ Mean value of the IC_50_ of compounds after 24 h stimulation, *n* = 6.

### 2.3 Analysis of the structure and activity of derivatives

**SCHEME 3:**
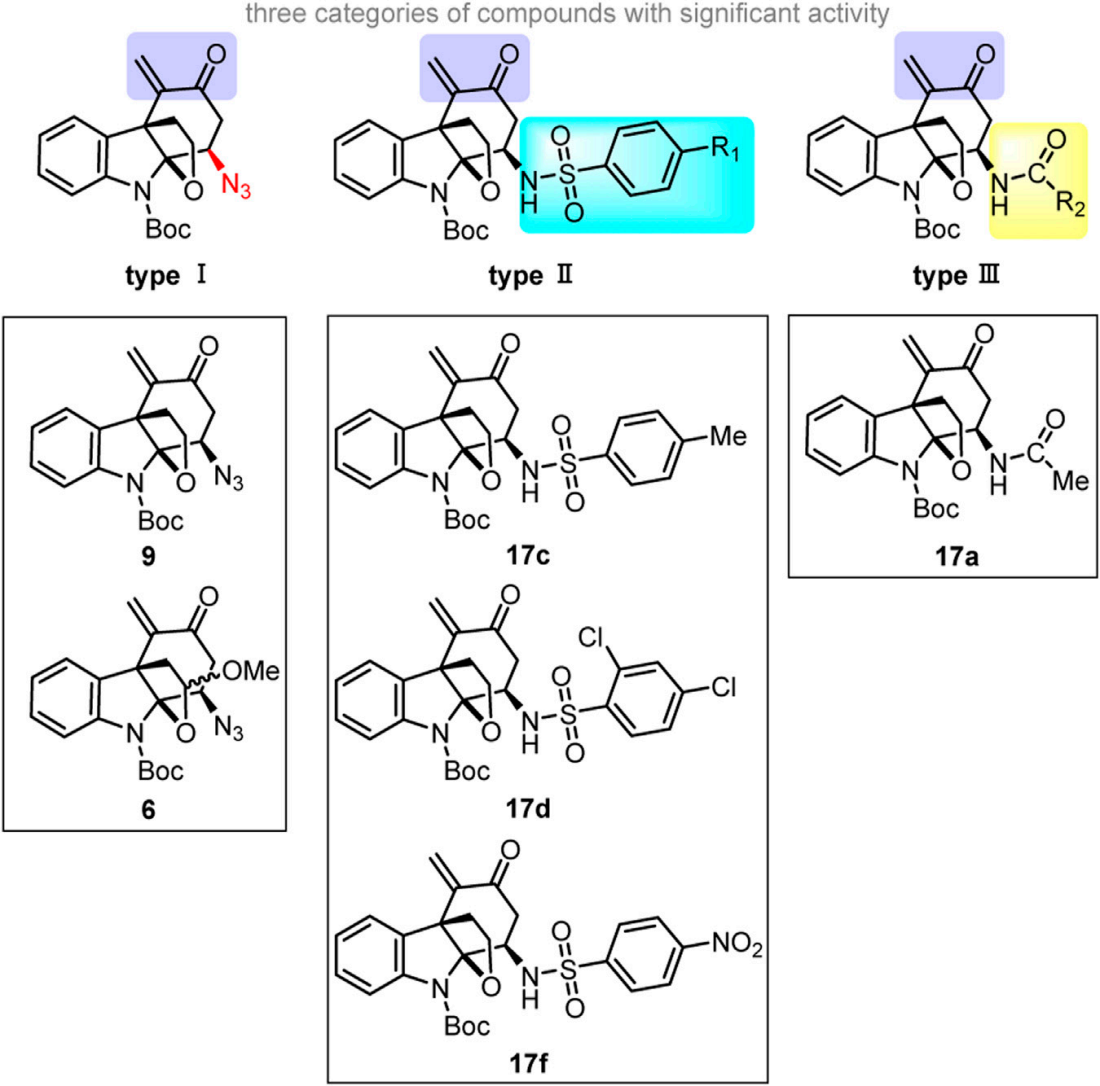
The skeletons of three types of effective derivatives and six specific lead compounds.

From a structural perspective, the six alkaloid derivatives that exhibited significant biological activity can be divided into three categories (Scheme 3). All these compounds have a unique tetracyclic core and an enone moiety.

As for type I derivatives, the difference in their inhibitory effect on the proliferation of RA-FLSs may correlate with the azido substituent on the cyclohexane ring in addition to the conjugated enone moiety. Compounds **6** and **9** showed significant inhibition of proliferation in MH7A cells, whereas azides **7**, **8**, **11**, and **13** with an α,β-unsaturated aldehyde or ester moiety had no obvious inhibition effect, suggesting that the combination of the azido group and the α,β-unsaturated ketone is critical to the enhancement of the inhibitory effect on the proliferation of RA-FLSs. (Arshad et al., 2017; Zhang et al., 2022b; Zhou et al., 2022). Among the amides/sulfonamides (see the structure of **17**), compound **17c**, a *para*-toluenesulfonamide, displayed the strongest inhibitory effect on the proliferation of RA-FLSs. (Zhao et al., 2016; Zhao et al., 2019). Moreover, the presence of one strong electron-donating group (**17e**) or one or more electron-withdrawing groups (**17d**, **17f**) on the benzene ring within the sulfonamide moiety greatly decreased the inhibitory effect on the proliferation of RA-FLSs.

## 3 Methods

### 3.1 Chemistry synthesis

#### 3.1.1 Synthesis of *tert*-butyl (4bS,8S,8aR)-8-azido-5-methylene-6-oxo-5,6,7,8-tetrahydro-9H-8a,4b-(epoxyethano) carbazole-9-carboxylate (9)

After a solution of **3** (108 mg, 0.28 mmol) was dissolved in dry DCM (5.0 mL), DMP (196.1 mg, 0.42 mmol) and NaHCO_3_ (47.1 mg, 0.56 mmol) were added to it at 0°C. The resultant mixture was stirred for 2 h at the same temperature. Then, the saturated aqueous Na_2_S_2_O_3_ (10 mL) was added to the mixture. The mixture was stirred for 0.5 h at room temperature and extracted with DCM (3 × 10 mL). The combined organic layer was washed with brine and then dried over Na_2_SO_4_. The solvent was removed under vacuum, and the residue was purified by column chromatography (petroleum ether/ethyl acetate = 4:1) to afford **9** (91.3 mg, 85%) as a white solid. ^**1**^ **H NMR** (400 MHz, CDCl_3_) *δ* 7.72–7.65 (m, 1H), 7.20 (ddd, J = 8.5, 6.2, 2.6 Hz, 1H), 7.05–6.95 (m, 2H), 6.21 (s, 1H), 5.64 (s, 1H), 5.34–5.29 (m, 1H), 4.32 (ddd, J = 8.6, 7.4, 1.1 Hz, 1H), 3.76 (ddd, J = 11.9, 9.1, 4.9 Hz, 1H), 2.66–2.51 (m, 2H), 2.48 (d, J = 4.7 Hz, 1H), 2.35 (dd, J = 17.4, 2.4 Hz, 1H), 1.62 (s, 9H). ^**13**^ **C NMR** (101 MHz, CDCl_3_) *δ* 195.5, 151.8, 146.9, 142.4, 130.5, 129.1, 124.1, 123.9, 120.5, 115.0, 105.2, 82.9, 67.8, 60.0, 58.0, 42.3, 39.1, 28.3. **HRMS (ESI)**: C_20_H_22_N_4_NaO_4_^+^ [(M + Na)^+^]: calcd: 405.1533; found: 405.1531.

#### 3.1.2 Synthesis of (4bS,8S,8aR)-8-azido-5-methylene-7,8-dihydro-9H-8a,4b-(epoxyethano) carbazol-6(5H)-one (10)

After a solution of **9** (91 mg, 0.24 mmol) was dissolved in dry DCM (5.0 mL), TMSOTf (125 μL, 0.72 mmol) was added at 0°C. After being stirred for 20 min, the reaction mixture was quenched with saturated NaHCO_3_ and extracted with EtOAc. The combined organic layers were washed with brine, dried over Na_2_SO_4_, and concentrated. The residue was purified by flash column chromatography (petroleum ether/ethyl acetate = 4:1) to give **10** (26.8 mg, 40%) as a white solid. ^**1**^ **H NMR** (400 MHz, CDCl_3_) *δ* 7.09 (td, J = 7.6, 1.3 Hz, 1H), 7.01 (dd, J = 7.6, 1.3 Hz, 1H), 6.80 (td, J = 7.5, 1.0 Hz, 1H), 6.61 (d, J = 7.8 Hz, 1H), 6.23 (d, J = 0.8 Hz, 1H), 5.68 (s, 1H), 4.42 (s, 1H), 4.29 (ddd, J = 8.8, 4.9, 3.0 Hz, 1H), 4.17 (dd, J = 4.9, 2.8 Hz, 1H), 3.82–3.68 (m, 1H), 2.77 (dd, J = 17.3, 2.9 Hz, 1H), 2.62 (dd, J = 17.3, 4.9 Hz, 1H), 2.55–2.48 (m, 2H).^**13**^ **C NMR** (101 MHz, CDCl_3_) *δ* 196.4, 148.0, 146.9, 129.7, 129.1, 124.2, 121.5, 120.28, 108.7, 103.0, 68.0, 60.5, 60.0, 43.0, 40.0. **HRMS (ESI)**: C_15_H_15_N_4_O_2_^+^ [(M + H)^+^]: calcd: 283.1190; found: 283.1189.

#### 3.1.3 Synthesis of 9-(*tert*-butyl) 5-methyl (4bS,8S,8aR)-8-azido-7,8-dihydro-9H-8a,4b-(epoxyethano) carbazole-5,9-dicarboxylate (13)

PTSA (104.6 mg, 0.55 mmol) was added to a solution of **3** (2.1 g, 5.5 mmol) in MeOH (20 mL). The resultant mixture was heated to reflux in an oil bath for 15 min, and then the solvent was removed *in vacuo* and the residue was dissolved in DCM (20 mL). PCC (2.4 g, 11 mmol), PTSA (1.0 g, 5.5 mmol), and silica gel (2.4 g) were added to the above mixture and then heated to reflux in an oil bath for 15 min. The mixture was filtered, and the filtrate was concentrated *in vacuo*. The residue was purified by column chromatography (petroleum ether/ethyl acetate = 15:1) to afford **11** (1.5 g, 63%, dr = 2:1, determined by crude ^1^H NMR) as a white foam.

After a solution of unsaturated aldehyde **11** (1.3 g, 3.4 mmol) was dissolved in *t*-BuOH (10 mL) and 2-methyl-2-butene (6 mL) at 0°C before a solution of sodium chlorite (3.1 g, 34.0 mmol) and monobasic sodium phosphate (4.1 g, 34.0 mmol) in H_2_O (10 mL) was added to it. The reaction was warmed to room temperature. After vigorous stirring for 4 h, the reaction mixture was poured into water (30 mL) and the resulting mixture was diluted with EtOAc (3 × 20 mL). The layers were separated, and the aqueous layer was extracted with EtOAc. The organic layers were combined and dried over Na_2_SO_4_, and concentrated *in vacuo* to produce a crude oil, which would be used for the next step without further purification.

After a solution of the acid **12** obtained from above in PhMe/MeOH (30 mL/3 mL) was cooled to 0°C, a solution of TMSCHN_2_ in hexanes (2.1 M, 3.4 mL) was added dropwise. The mixture was warmed to room temperature and stirred for 0.5 h before being quenched with silica gel. The solvent was directly removed *in vacuo* and the residue was then purified by flash column chromatography on silica gel (petroleum ether/ethyl acetate = 30:1) to give **13** (1.40 g, 69% over two steps) as a white solid. ^**1**^ **H NMR** (400 MHz, CDCl_3_) *δ* 7.70 (d, J = 8.3 Hz, 1H), 7.61 (ddd, J = 7.7, 1.5, 0.6 Hz, 1H), 7.20 (ddd, J = 8.2, 7.4, 1.5 Hz, 1H), 6.98 (td, J = 7.5, 1.1 Hz, 1H), 6.87 (td, J = 4.7, 0.8 Hz, 1H), 5.46 (s, 1H), 3.69 (ddd, J = 12.3, 8.6, 4.9 Hz, 1H), 3.07 (dd, J = 12.6, 4.9 Hz, 1H), 2.56 (td, J = 12.5, 7.9 Hz, 1H), 2.43 (dd, J = 4.6, 3.1 Hz, 2H), 1.63 (s, 9H). ^**13**^ **C NMR** (101 MHz, CDCl_3_) *δ* 165.7, 152.3, 142.2, 135.3, 134.5, 132.0, 128.6, 124.8, 123.4, 115.4, 105.2, 82.8, 68.8, 57.7, 57.6, 51.7, 39.2, 28.5, 28.3. **HRMS (ESI)**: C_21_H_24_N_4_NaO_5_^+^ [(M + Na)^+^]: calcd: 435.1639; found: 435.1642.

#### 3.1.4 Synthesis of *tert*-butyl (4bS,8S,8aR)-8-azido-11-methoxy-5-methylene-6-oxo-5,6,7,8-tetrahydro-9H-8a,4b-(epoxyethano) carbazole-9-carboxylate (6)

After a solution of **5** (50.2 mg, 0.12 mmol) was dissolved in dry DCM (4.0 mL), DMP (84 mg, 0.18 mmol) and NaHCO_3_ (20.1 mg, 0.24 mmol) were added to it at 0 °C. The resultant mixture was stirred for 2h at the same temperature. Then, the saturated aqueous Na_2_S_2_O_3_ (15 mL) was added to the mixture. The mixture was stirred for 0.5 h at room temperature and extracted with DCM (3 × 10 mL). Then, the combined organic layers were washed with brine and then dried over Na_2_SO_4_. The solvent was removed under vacuum and the residue was purified by column chromatography (petroleum ether/ethyl acetate = 4:1) to afford **6** (43 mg, 86%) as a white solid. ^**1**^ **H NMR** (400 MHz, CDCl_3_) *δ* 7.66 (s, 1H), 7.18 (ddd, J = 8.5, 4.9, 3.8 Hz, 1H), 6.99 (dd, J = 4.0, 0.9 Hz, 2H), 6.18 (s, 1H), 5.58 (s, 1H), 5.42 (dd, J = 3.3, 2.2 Hz, 1H), 5.37 (s, 1H), 3.23 (s, 3H), 2.72–2.66 (m, 2H), 2.58 (dd, J = 17.6, 4.3 Hz, 1H), 2.40 (dd, J = 17.6, 2.4 Hz, 1H), 1.65 (s, 9H). ^**13**^ **C NMR** (101 MHz, CDCl_3_) *δ* 195.1, 151.7, 147.3, 132.0, 128.9, 123.8, 123.7, 120.3, 115.4, 106.8, 105.3, 82.8, 55.0, 48.1, 38.9, 28.4. **HRMS (ESI)**: C_21_H_24_N_4_NaO_5_^+^ [(M + Na)^+^]: calcd: 435.1639; found: 435.1640.

#### 3.1.5 Synthesis of (compound 17a-17f) *tert*-butyl (4bS,8S,8aR)-5-methylene-8-((4-methylphenyl) sulfonamido)-6-oxo-5,6,7,8-tetrahydro-9H-8a,4b-(epoxyethano) carbazole-9-carboxylate (17c)

After a solution of compound **3** (210 mg, 0.55 mmol) was dissolved in DCM (5 mL), 2,6-lutidine (128 uL, 1.10 mmol) and TBSOTf (150 uL, 0.66 mmol) were added to it at 0 °C and stirred for 0.5 h. Then the mixture was diluted with DCM (3 × 10 mL), washed with H_2_O three times, dried over Na_2_SO_4_, filtered, and concentrated under a vacuum. The crude product was dissolved in THF (30 mL) and then Ph_3_P (430 mg, 1.65 mmol) and H_2_O (0.1 mL, 5.5 mmol) were added. The reaction mixture was heated at 50 °C for 12 h. The solvent was directly removed *in vacuo* and the residue was then purified by flash column chromatography (DCM/MeOH = 50:1). The crude product was directly dissolved in DCM (3 mL), and then triethylamine (160 uL, 1.1 mmol) and 4-Methylbenzenesulfonyl chloride (116 mg, 0.61 mmol) were added sequentially at 0°C. The resulting solution was warmed to room temperature and stirred for 2 h and then was quenched with NH_4_Cl (aq.). The aqueous phase was extracted with DCM. The combined organic layers were washed with brine and then dried over Na_2_SO_4_. The solvent was removed *in vacuo* and the residue was then purified by flash column chromatography (petroleum ether/ethyl acetate = 5:1) to give **16c** (134.5 mg, 39% over three steps) as a white solid.

After a solution of **16c** (134 mg, 0.21 mmol) was dissolved in THF (3 mL), Tetrabutylammonium fluoride (1.0 M in THF, 1 mL, 1.05 mmol) was added. The resulting mixture was stirred for 24 h at 60°C and then was quenched with NH_4_Cl (aq.). After the reaction was terminated, the aqueous layer was extracted with EtOAc. The combined organic layers were washed with H_2_O and brine, dried over anhydrous Na_2_SO_4_, and concentrated *in vacuo* to produce a crude oil, which was purified by column chromatography (petroleum ether/ethyl acetate = 2:1). The crude product was directly dissolved in DCM (3 mL), and then DMP (147 mg, 0.32 mmol) and NaHCO_3_ (35.3 mg, 0.42 mmol) were added sequentially at 0°C. The resultant mixture was stirred for 2 h at the same temperature. Then, the saturated aqueous Na_2_S_2_O_3_ (10 mL) was added to the mixture. The mixture was stirred for 0.5 h at room temperature and extracted with DCM (3 × 5 mL). The combined organic layer was washed with brine, then dried over Na_2_SO_4_. The solvent was removed under vacuum, and the residue was purified by column chromatography (petroleum ether/ethyl acetate = 4:1) to afford **17c** (83.6 mg, 78% over two steps) as a white solid. ^**1**^ **H NMR** (400 MHz, CDCl_3_) *δ* 7.74 (d, J = 8.0 Hz, 2H), 7.65 (d, J = 8.2 Hz, 1H), 7.31 (d, J = 8.0 Hz, 2H), 7.16 (ddd, J = 8.5, 6.5, 2.3 Hz, 1H), 7.00–6.90 (m, 2H), 6.15 (s, 1H), 5.56 (s, 1H), 5.24 (s, 1H), 4.74 (dt, J = 3.9, 1.8 Hz, 1H), 4.16 (dd, J = 9.2, 7.3 Hz, 1H), 3.65 (ddd, J = 12.1, 9.2, 4.8 Hz, 1H), 2.95 (dd, J = 17.9, 4.3 Hz, 1H), 2.49 (dt, J = 12.1, 6.0 Hz, 1H), 2.43 (s, 3H), 2.33 (dd, J = 11.9, 4.7 Hz, 1H), 2.25 (dt, J = 17.9, 1.7 Hz, 1H), 1.41 (s, 9H). ^**13**^ **C NMR** (101 MHz, CDCl_3_) *δ* 195.5, 151.4, 146.6, 143.8, 142.6, 136.2, 130.2, 129.7, 129.1, 127.3, 124.2, 123.9, 120.2, 114.9, 103.5, 82.7, 67.6, 60.2, 51.1, 42.0, 38.2, 28.0, 21.5. **HRMS (ESI)**: C_27_H_30_N_2_NaO_6_S^+^ [(M + H)^+^]: calcd: 533.1717; found: 533.1720.

#### 3.1.6 Synthesis of *tert*-butyl (4bS,8S,8aR)-8-acetamido-5-methylene-6-oxo-5,6,7,8-tetrahydro-9H-8a,4b-(epoxyethano) carbazole-9-carboxylate (17a)

The product of **15** (260 mg, 0.55 mmol) was directly dissolved in DCM (3 mL), and then triethylamine (160 uL, 1.1 mmol) and acetyl chloride (43.5 μL, 0.61 mmol) were added sequentially at 0°C. The resulting solution was warmed to room temperature and stirred for 2 h and then was quenched with NH_4_Cl (aq.). The aqueous phase was extracted with DCM. The combined organic layers were washed with brine and then dried over Na_2_SO_4_. The solvent was removed *in vacuo* and the residue was then purified by flash column chromatography (petroleum ether/ethyl acetate = 5:1) to give **16a** (249 mg, 88%) as a white solid.

After a solution of **16a** (108 mg, 0.21 mmol) was dissolved in THF (3 mL), Tetrabutylammonium fluoride (1.0 M in THF, 1 mL, 1.05 mmol) was added. The resulting mixture was stirred for 24 h at 60°C and then was quenched with NH_4_Cl (aq.). After the reaction was terminated, the aqueous layer was extracted with EtOAc. The combined organic layers were washed with H_2_O and brine, dried over anhydrous Na_2_SO_4_, and concentrated *in vacuo* to produce a crude oil, which was purified by column chromatography (petroleum ether/ethyl acetate = 2:1). The crude product was directly dissolved in DCM (3 mL), and then DMP (147 mg, 0.32 mmol) and NaHCO_3_ (35.3 mg, 0.42 mmol) were added sequentially at 0°C. The resultant mixture was stirred for 2 h at the same temperature. Then, the saturated aqueous Na_2_S_2_O_3_ (10 mL) was added to the mixture. The mixture was stirred for 0.5 h at room temperature and extracted with DCM (3 × 5 mL). The combined organic layer was washed with brine, then dried over Na_2_SO_4_. The solvent was removed under vacuum, and the residue was purified by column chromatography (petroleum ether/ethyl acetate = 4:1) to afford **17a** (54.5 mg, 65% over two steps) as a white solid. ^**1**^ **H NMR** (400 MHz, CDCl_3_) *δ* 7.70 (d, J = 8.3 Hz, 1H), 7.20 (ddd, J = 8.4, 6.5, 2.3 Hz, 1H), 7.02–6.92 (m, 2H), 6.29 (s, 1H), 5.67 (d, J = 5.8 Hz, 2H), 5.39 (q, J = 2.0 Hz, 1H), 4.30–4.21 (m, 1H), 3.75 (ddd, J = 11.8, 9.4, 5.2 Hz, 1H), 3.07 (dd, J = 17.6, 4.4 Hz, 1H), 2.53–2.35 (m, 2H), 2.32 (dd, J = 17.6, 2.6 Hz, 1H), 1.93 (s, 3H), 1.59 (s, 9H). ^**13**^ **C NMR** (101 MHz, CDCl_3_) *δ* 196.5, 170.2, 151.4, 146.6, 142.8, 130.1, 129.3, 124.1, 123.7, 121.3, 115.0, 104.1, 82.9, 67.2, 60.2, 48.6, 42.4, 38.9, 28.3, 23.5. **HRMS (ESI)**: C_22_H_26_N_2_NaO_5_^+^ [(M + Na)^+^]: calcd: 421.1734; found: 421.1737.

#### 3.1.7 Synthesis of *tert*-butyl (4bS,8S,8aR)-5-methylene-6-oxo-8-(2,2,2-trifluoroacetamido)-5,6,7,8-tetrahydro-9H-8a,4b-(epoxyethano) carbazole-9-carboxylate (17b)

The product of **15** (260 mg, 0.55 mmol) was directly dissolved in DCM (3 mL), and then triethylamine (160 uL, 1.1 mmol) and 2,2,2-trifluoroacetyl chloride (54 μL, 0.61 mmol) were added sequentially at 0°C. The resulting solution was warmed to room temperature and stirred for 2 h and then was quenched with NH_4_Cl (aq.). The aqueous phase was extracted with DCM. The combined organic layers were washed with brine and then dried over Na_2_SO_4_. The solvent was removed *in vacuo* and the residue was then purified by flash column chromatography (petroleum ether/ethyl acetate = 5:1) to give **16b** (263 mg, 84%) as a white solid.

After a solution of **16b** (119.5 mg, 0.21 mmol) was dissolved in THF (3 mL), Tetrabutylammonium fluoride (1.0 M in THF, 1 mL, 1.05 mmol) was added. The resulting mixture was stirred for 24 h at 60°C and then was quenched with NH_4_Cl (aq.). After the reaction was terminated, the aqueous layer was extracted with EtOAc. The combined organic layers were washed with H_2_O and brine, dried over anhydrous Na_2_SO_4_, and concentrated *in vacuo* to produce a crude oil, which was purified by column chromatography (petroleum ether/ethyl acetate = 2:1). The crude product was directly dissolved in DCM (3 mL), and then DMP (147 mg, 0.32 mmol) and NaHCO_3_ (35.3 mg, 0.42 mmol) were added sequentially at 0°C. The resultant mixture was stirred for 2 h at the same temperature. Then, the saturated aqueous Na_2_S_2_O_3_ (10 mL) was added to the mixture. The mixture was stirred for 0.5 h at room temperature and extracted with DCM (3 × 5 mL). The combined organic layer was washed with brine, then dried over Na_2_SO_4_. The solvent was removed under vacuum, and the residue was purified by column chromatography (petroleum ether/ethyl acetate = 4:1) to afford **17b** (59 mg, 62% over two steps) as a white solid. ^**1**^ **H NMR** (400 MHz, CDCl_3_) *δ* 7.68 (d, J = 8.2 Hz, 1H), 7.22 (ddd, J = 8.5, 6.5, 2.3 Hz, 1H), 7.05–6.94 (m, 2H), 6.70 (s, 1H), 6.29 (s, 1H), 5.71 (s, 1H), 5.40 (td, J = 4.9, 2.9 Hz, 1H), 4.35–4.24 (m, 1H), 3.84–3.72 (m, 1H), 3.08 (dd, J = 17.9, 4.7 Hz, 1H), 2.51–2.39 (m, 3H), 1.59 (s, 9H). ^**13**^ **C NMR** (101 MHz, CDCl_3_) *δ* 195.3, 157.5, 157.1, 156.8, 156.4, 151.4, 146.1, 142.4, 129.6, 129.4, 124.1, 124.0, 122.3, 119.7, 116.8, 115.1, 113.9, 111.1, 103.4, 83.4, 67.5, 60.3, 49.2, 42.3, 37.9, 28.2. ^**19**^ **F NMR** (376 MHz, CDCl_3_) *δ* -76.0. **HRMS (ESI)**: C_22_H_23_F_3_N_2_NaO_5_^+^ [(M + Na)^+^]: calcd: 475.1451; found: 475.1452.

#### 3.1.8 Synthesis of *tert*-butyl (4bS,8S,8aR)-8-((2,4-dichlorophenyl) sulfonamido)-5-methylene-6-oxo-5,6,7,8-tetrahydro-9H-8a,4b-(epoxyethano) carbazole-9-carboxylate (17d)

The product of **15** (260 mg, 0.55 mmol) was directly dissolved in DCM (3 mL), and then triethylamine (160 uL, 1.1 mmol) and 2,4-dichlorobenzenesulfonyl chloride (150 mg, 0.61 mmol) were added sequentially at 0°C. The resulting solution was warmed to room temperature and stirred for 2 h and then was quenched with NH_4_Cl (aq.). The aqueous phase was extracted with DCM. The combined organic layers were washed with brine and then dried over Na_2_SO_4_. The solvent was removed *in vacuo* and the residue was then purified by flash column chromatography (petroleum ether/ethyl acetate = 5:1) to give **16d** (326 mg, 87%) as a white solid.

After a solution of **16d** (143 mg, 0.21 mmol) was dissolved in THF (3 mL), Tetrabutylammonium fluoride (1.0 M in THF, 1 mL, 1.05 mmol) was added. The resulting mixture was stirred for 24 h at 60°C and then was quenched with NH_4_Cl (aq.). After the reaction was terminated, the aqueous layer was extracted with EtOAc. The combined organic layers were washed with H_2_O and brine, dried over anhydrous Na_2_SO_4_, and concentrated *in vacuo* to produce a crude oil, which was purified by column chromatography (petroleum ether/ethyl acetate = 2:1). The crude product was directly dissolved in DCM (3 mL), and then DMP (147 mg, 0.32 mmol) and NaHCO_3_ (35.3 mg, 0.42 mmol) were added sequentially at 0°C. The resultant mixture was stirred for 2 h at the same temperature. Then, the saturated aqueous Na_2_S_2_O_3_ (10 mL) was added to the mixture. The mixture was stirred for 0.5 h at room temperature and extracted with DCM (3 × 5 mL). The combined organic layer was washed with brine, then dried over Na_2_SO_4_. The solvent was removed under vacuum, and the residue was purified by column chromatography (petroleum ether/ethyl acetate = 4:1) to afford **17d** (89 mg, 75% over two steps) as a white solid. ^**1**^ **H NMR** (400 MHz, CDCl_3_) *δ* 8.01 (d, J = 8.5 Hz, 1H), 7.65 (d, J = 8.3 Hz, 1H), 7.53 (d, J = 2.0 Hz, 1H), 7.42 (dd, J = 8.5, 2.0 Hz, 1H), 7.17 (ddd, J = 8.5, 6.9, 1.9 Hz, 1H), 7.02–6.90 (m, 2H), 6.17–6.12 (m, 1H), 5.63 (s, 1H), 5.50 (d, J = 2.7 Hz, 1H), 4.97–4.90 (m, 1H), 4.10 (dd, J = 9.3, 7.3 Hz, 1H), 3.67 (ddd, J = 12.2, 9.3, 4.8 Hz, 1H), 2.85 (dd, J = 18.0, 4.1 Hz, 1H), 2.50 (td, J = 12.0, 7.4 Hz, 1H), 2.38 (dd, J = 11.9, 4.7 Hz, 1H), 2.34–2.24 (m, 1H), 1.49 (s, 9H). ^**13**^ **C NMR** (101 MHz, CDCl_3_) *δ* 195.4, 151.3, 146.4, 142.6, 139.7, 135.9, 132.8, 132.0, 131.3, 129.9, 129.3, 127.5, 124.2, 124.0, 120.9, 115.1, 103.4, 83.0, 67.7, 60.2, 51.5, 42.1, 38.7, 28.2. **HRMS (ESI)**: C_26_H_26_Cl_2_N_2_NaO_6_S^+^ [(M + Na)^+^]: calcd: 587.0781; found: 587.0781.

#### 3.1.9 Synthesis of *tert*-butyl (4bS,8S,8aR)-8-((4-methoxyphenyl) sulfonamido)-5-methylene-6-oxo-5,6,7,8-tetrahydro-9H-8a,4b-(epoxyethano) carbazole-9-carboxylate (17e)

The product of **15** (260 mg, 0.55 mmol) was directly dissolved in DCM (3 mL), and then triethylamine (160 uL, 1.1 mmol) and 4-Methoxybenzenesulfonyl chloride (126 mg, 0.61 mmol) were added sequentially at 0°C. The resulting solution was warmed to room temperature and stirred for 2 h and then was quenched with NH_4_Cl (aq.). The aqueous phase was extracted with DCM. The combined organic layers were washed with brine and then were dried over Na_2_SO_4_. The solvent was removed *in vacuo* and the residue was then purified by flash column chromatography (petroleum ether/ethyl acetate = 5:1) to give **16e** (328 mg, 93%) as a white solid.

After a solution of **16e** (135 mg, 0.21 mmol) was dissolved in THF (3 mL), Tetrabutylammonium fluoride (1.0 M in THF, 1 mL, 1.05 mmol) was added. The resulting mixture was stirred for 24 h at 60°C and then was quenched with NH_4_Cl (aq.). After the reaction was terminated, the aqueous layer was extracted with EtOAc. The combined organic layers were washed with H_2_O and brine, dried over anhydrous Na_2_SO_4_, and concentrated *in vacuo* to produce a crude oil, which was purified by column chromatography (petroleum ether/ethyl acetate = 2:1). The crude product was directly dissolved in DCM (3 mL), and then DMP (147 mg, 0.32 mmol) and NaHCO_3_ (35.3 mg, 0.42 mmol) were added sequentially at 0°C. The resultant mixture was stirred for 2 h at the same temperature. Then, the saturated aqueous Na_2_S_2_O_3_ (10 mL) was added to the mixture. The mixture was stirred for 0.5 h at room temperature and extracted with DCM (3 × 5 mL). The combined organic layer was washed with brine, then dried over Na_2_SO_4_. The solvent was removed under vacuum, and the residue was purified by column chromatography (petroleum ether/ethyl acetate = 4:1) to afford **17e** (80 mg, 72% over two steps) as a white solid. ^**1**^ **H NMR** (400 MHz, CDCl_3_) *δ* 7.83–7.74 (m, 2H), 7.66 (d, J = 8.2 Hz, 1H), 7.17 (ddd, J = 8.5, 6.6, 2.2 Hz, 1H), 7.02–6.91 (m, 4H), 6.16 (s, 0H), 5.57 (s, 1H), 5.11 (s, 1H), 4.77 (dt, J = 3.8, 1.7 Hz, 1H), 4.19 (dd, J = 9.3, 7.3 Hz, 1H), 3.88 (s, 3H), 3.68 (ddd, J = 12.2, 9.3, 4.8 Hz, 1H), 2.94 (dd, J = 17.9, 4.2 Hz, 1H), 2.49 (td, J = 12.1, 7.4 Hz, 1H), 2.36 (dd, J = 11.9, 4.7 Hz, 1H), 2.25 (ddd, J = 17.8, 2.1, 1.2 Hz, 1H), 1.45 (s, 9H). ^**13**^ **C NMR** (101 MHz, CDCl_3_) *δ* 195.4, 163.2, 151.4, 146.7, 142.6, 130.8, 130.2, 129.5, 129.2, 124.2, 124.0, 120.2, 115.0, 114.4, 103.5, 82.8, 67.6, 60.2, 55.6, 51.2, 42.2, 38.1, 28.1. **HRMS (ESI)**: C_27_H_30_N_2_NaO_7_S^+^ [(M + Na)^+^]: calcd: 549.1666; found: 549.1669.

#### 3.1.10 Synthesis of *tert*-butyl(4bS,8S,8aR)-5-methylene-8-((4-nitrophenyl) sulfonamido)-6-oxo-5,6,7,8-tetrahydro-9H-8a,4b-(epoxyethano) carbazole-9-carboxylate (17f)

The product of **15** (260 mg, 0.55 mmol) was directly dissolved in DCM (3 mL), and then triethylamine (160 uL, 1.1 mmol) and 4-nitrobenzenesulfonyl chloride (135 mg, 0.61 mmol) were added sequentially at 0 °C. The resulting solution was warmed to room temperature and stirred for 2 h and then was quenched with NH_4_Cl (aq.). The aqueous phase was extracted with DCM. The combined organic layers were washed with brine and then dried over Na_2_SO_4_. The solvent was removed *in vacuo* and the residue was then purified by flash column chromatography (petroleum ether/ethyl acetate = 5:1) to give **16f** (326 mg, 90%) as a white solid.

After a solution of **16f** (138 mg, 0.21 mmol) was dissolved in THF (3 mL), Tetrabutylammonium fluoride (1.0 M in THF, 1 mL, 1.05 mmol) was added. The resulting mixture was stirred for 24 h at 60 °C and then was quenched with NH_4_Cl (aq.). After the reaction was terminated, the aqueous layer was extracted with EtOAc. The combined organic layers were washed with H_2_O and brine, dried over anhydrous Na_2_SO_4_, and concentrated *in vacuo* to produce a crude oil, which was purified by column chromatography (petroleum ether/ethyl acetate = 2:1). The crude product was directly dissolved in DCM (3 mL), and then DMP (147 mg, 0.32 mmol) and NaHCO_3_ (35.3 mg, 0.42 mmol) were added sequentially at 0 °C. The resultant mixture was stirred for 2 h at the same temperature. Then, the saturated aqueous Na_2_S_2_O_3_ (10 mL) was added to the mixture. The mixture was stirred for 0.5 h at room temperature and extracted with DCM (3 × 5 mL). The combined organic layer was washed with brine, then dried over Na_2_SO_4_. The solvent was removed under vacuum, and the residue was purified by column chromatography (petroleum ether/ethyl acetate = 4:1) to afford **17f** (80 mg, 70% over two steps) as a white solid. ^**1**^ **H NMR** (400 MHz, CDCl_3_) *δ* 8.38 (d, J = 8.9 Hz, 2H), 8.06 (d, J = 8.9 Hz, 2H), 7.60 (d, J = 8.3 Hz, 1H), 7.19 (ddd, J = 8.5, 7.1, 1.7 Hz, 1H), 7.03–6.91 (m, 2H), 6.18 (s, 1H), 5.63 (s, 1H), 5.38–5.33 (m, 1H), 5.00 (dt, J = 4.4, 2.2 Hz, 1H), 4.20–4.11 (m, 1H), 3.69 (ddd, J = 12.0, 9.3, 5.0 Hz, 1H), 2.90 (dd, J = 17.8, 4.2 Hz, 1H), 2.47 (td, J = 12.0, 7.4 Hz, 1H), 2.40–2.35 (m, 1H), 2.32 (ddd, J = 17.8, 2.3, 1.0 Hz, 1H), 1.52 (s, 9H). ^**13**^ **C NMR** (101 MHz, CDCl_3_) *δ* 195.0, 151.2, 150.2, 146.2, 145.2, 142.3, 129.9, 129.4, 128.7, 124.3, 124.2, 124.1, 121.3, 115.0, 103.3, 83.3, 67.6, 60.1, 51.3, 42.3, 38.8, 28.2. **HRMS (ESI)**: C_26_H_27_N_3_NaO_8_S^+^ [(M + Na)^+^]: calcd: 567.1411; found: 567.1412.

#### 3.1.11 Synthesis of (compound 20a-20c): *tert*-butyl (4bS,8S,8aR)-8-(2-(4-(4-methoxyphenyl)-1H-1,2,3-triazol-1-yl) acetamido)-5-methylene-6-oxo-5,6,7,8-tetrahydro-9H-8a,4b-(epoxyethano) carbazole-9-carboxylate (20a)

After a solution of compound **15** (1.3 g, 2.75 mmol) was dissolved in DCM (20 mL), triethylamine (0.8 mL, 5.5 mmol) and chloroacetyl chloride (239 uL, 3.0 mmol) were added at 0°C. Having been stirred for 2 h, the mixture was diluted with DCM (3 × 20 mL), washed with H_2_O three times, dried over Na_2_SO_4_, filtered, and concentrated under a vacuum. The crude product was dissolved in DMF (12 mL) and H_2_O (4 mL) and then NaN_3_ (536 mg, 8.25 mmol) was added. The reaction mixture was heated at room temperature for 24 h. The solvent was directly removed *in vacuo* and the residue was then purified by flash column chromatography (DCM/MeOH = 100:1) to give **18** (978 mg, 64%, over two steps) as a white solid.

After a solution of **18** (86 mg, 0.15 mmol) was dissolved in *t*-BuOH/H_2_O (2 mL/2 mL), sodium ascorbate (6.0 mg, 0.03 mmol), CuSO_4_·H_2_O (5.4 mg, 0.03 mmol) and 1-ethynyl-4-methoxybenzene (22 mg, 0.17 mmol) were added at room temperature. After a vigorous stirring for 3 h, the resulting mixture was diluted with EtOAc (3 × 5 mL). The layers were separated, and the aqueous layers were extracted with EtOAc. The organic layers were combined and dried over Na_2_SO_4_, concentrated *in vacuo*, and then purified by flash column chromatography (petroleum ether/ethyl acetate = 5:1) to give **19a** (72 mg, 68%, over two steps) as a white solid.

After a solution of **19a** (72 mg, 0.105 mmol) was dissolved in THF (3 mL), tetrabutylammonium fluoride (1.0 M in THF, 0.5 mL, 0.53 mmol) was added, and the resulting mixture was stirred for 24 h at 60°C, and then was quenched with NH_4_Cl (aq.). After the reaction was terminated, the aqueous layer was extracted with EtOAc. The combined organic layers were washed with H_2_O and brine, dried over anhydrous Na_2_SO_4_, and concentrated *in vacuo* to produce a crude oil, which was purified by column chromatography (petroleum ether/ethyl acetate = 2:1). The crude product was directly dissolved in DCM (3 mL), and then DMP (74 mg, 0.16 mmol) and NaHCO_3_ (18 mg, 0.21 mmol) were added sequentially at 0°C. The resultant mixture was stirred for 2 h at the same temperature. Then, the saturated aqueous Na_2_S_2_O_3_ (10 mL) was added to the mixture. The mixture was stirred for 0.5 h at room temperature and extracted with DCM (3 × 5 mL). The combined organic layers were washed with brine and then dried over Na_2_SO_4_. The solvent was removed under vacuum, and the residue was purified by column chromatography (petroleum ether/ethyl acetate = 4:1) to afford **20a** (39 mg, 65% over two steps) as a white solid. ^**1**^ **H NMR** (400 MHz, CDCl_3_) *δ* 7.81–7.72 (m, 3H), 7.66 (d, J = 8.3 Hz, 1H), 7.17 (ddd, J = 8.4, 7.3, 1.4 Hz, 1H), 7.04–6.98 (m, 2H), 6.94 (td, J = 7.5, 1.0 Hz, 1H), 6.86 (dd, J = 7.7, 1.5 Hz, 1H), 6.23 (d, J = 5.4 Hz, 1H), 5.97 (s, 1H), 5.53–5.45 (m, 1H), 5.40 (s, 1H), 5.12 (d, J = 16.5 Hz, 1H), 4.98 (d, J = 16.5 Hz, 1H), 4.14 (dd, J = 9.0, 7.3 Hz, 1H), 3.87 (s, 3H), 3.63 (ddd, J = 11.8, 9.2, 5.0 Hz, 1H), 3.01 (dd, J = 17.8, 4.1 Hz, 1H), 2.34 (dd, J = 17.8, 2.5 Hz, 1H), 2.28–2.13 (m, 2H), 1.60 (s, 9H). ^**13**^ **C NMR** (101 MHz, CDCl_3_) *δ* 196.2, 165.2, 160.0, 151.4, 148.6, 146.3, 142.7, 129.9, 129.3, 127.0, 124.1, 123.9, 122.4, 121.7, 120.3, 115.0, 114.5, 103.6, 83.1, 67.4, 60.1, 55.3, 53.4, 48.3, 41.9, 38.4, 29.7, 28.3. **HRMS (ESI)**: C_31_H_34_N_5_O_6_^+^ [(M + H)^+^]: calcd: 572.2504; found: 572.2505.

#### 3.1.12 Synthesis of *tert*-butyl (4bS,8S,8aR)-8-(2-(4-(4-fluorophenyl)-1H-1,2,3-triazol-1-yl) acetamido)-5-methylene-6-oxo-5,6,7,8-tetrahydro-9H-8a,4b-(epoxyethano) carbazole-9-carboxylate (20b)

After a solution of **18** (86 mg, 0.15 mmol) was dissolved in *t*-BuOH/H_2_O (2 mL/2 mL), sodium ascorbate (6.0 mg, 0.03 mmol), CuSO_4_·H_2_O (5.4 mg, 0.03 mmol) and 1-ethynyl-4-fluorobenzene (20.5 mg, 0.17 mmol) were added at room temperature. After a vigorous stirring for 3 h, the resulting mixture was diluted with EtOAc (3 × 5 mL). The layers were separated, and the aqueous layer was extracted with EtOAc. The organic layer was combined and dried over Na_2_SO_4_, concentrated *in vacuo*, and then purified by flash column chromatography (petroleum ether/ethyl acetate = 5:1) to give **19b** (73 mg, 72%, over two steps) as a white solid.

After a solution of **19b** (72 mg, 0.107 mmol) was dissolved in THF (3 mL), tetrabutylammonium fluoride (1.0 M in THF, 0.5 mL, 0.53 mmol) was added, and the resulting mixture was stirred for 24 h at 60°C, and then was quenched with NH_4_Cl (aq.). After the reaction was terminated, the aqueous layer was extracted with EtOAc. The combined organic layers were washed with H_2_O and brine, dried over anhydrous Na_2_SO_4_, and concentrated *in vacuo* to produce a crude oil, which was purified by column chromatography (petroleum ether/ethyl acetate = 2:1). The crude product was directly dissolved in DCM (3 mL), and then DMP (74 mg, 0.16 mmol) and NaHCO_3_ (18 mg, 0.21 mmol) were added sequentially at 0 °C. The resultant mixture was stirred for 2 h at the same temperature. Then, the saturated aqueous Na_2_S_2_O_3_ (10 mL) was added to the mixture. The mixture was stirred for 0.5 h at room temperature and extracted with DCM (3 × 5 mL). The combined organic layers were washed with brine, then dried over Na_2_SO_4_. The solvent was removed under vacuum, and the residue was purified by column chromatography (petroleum ether/ethyl acetate = 4:1) to afford **20b** (42 mg, 71% over two steps) as a white solid. ^**1**^ **H NMR** (400 MHz, CDCl_3_) *δ* 7.87–7.76 (m, 3H), 7.64 (d, J = 8.3 Hz, 1H), 7.16 (dtd, J = 8.8, 7.2, 1.8 Hz, 3H), 6.94 (td, J = 7.5, 1.0 Hz, 1H), 6.86 (dd, J = 7.7, 1.4 Hz, 1H), 6.30 (d, J = 5.3 Hz, 1H), 5.97 (s, 1H), 5.48 (td, J = 4.5, 2.4 Hz, 1H), 5.41 (s, 1H), 5.11 (d, J = 16.4 Hz, 1H), 4.99 (d, J = 16.4 Hz, 1H), 4.17–4.06 (m, 1H), 3.63 (ddd, J = 11.7, 9.2, 5.1 Hz, 1H), 3.01 (dd, J = 17.7, 4.2 Hz, 1H), 2.33 (dd, J = 17.8, 2.5 Hz, 2H), 2.29–2.15 (m, 1H), 1.58 (s, 9H). ^**13**^ **C NMR** (101 MHz, CDCl_3_) *δ* 196.1, 165.0, 164.1, 161.6, 151.4, 147.7, 146.3, 142.6, 129.8, 129.3, 127.5, 127.4, 126.1, 126.0, 124.1, 123.9, 121.6, 121.0, 116.2, 116.0, 115.0, 103.6, 83.1, 67.4, 60.0, 53.4, 48.4, 42.0, 38.4, 28.3. ^**19**^ **F NMR** (376 MHz, CDCl3) *δ* -112.5. **HRMS (ESI)**: C_30_H_31_FN_5_O_5_^+^ [(M + H)^+^]: calcd: 560.2304; found: 560.2308.

#### 3.1.13 Synthesis of *tert*-butyl (4bS,8S,8aR)-5-methylene-6-oxo-8-(2-(4-(pyridin-2-yl)-1H-1,2,3-triazol-1-yl) acetamido)-5,6,7,8-tetrahydro-9H-8a,4b-(epoxyethano) carbazole-9-carboxylate (20c)

After a solution of **18** (86 mg, 0.15 mmol) was dissolved in *t*-BuOH/H_2_O (2 mL/2 mL), sodium ascorbate (6.0 mg, 0.03 mmol), CuSO_4_·H_2_O (5.4 mg, 0.03 mmol) and 2-ethynylpyridine (17.5 mg, 0.17 mmol) were added at room temperature. After a vigorous stirring for 3 h, the resulting mixture was diluted with EtOAc (3 × 5 mL). The layers were separated, and the aqueous layer was extracted with EtOAc. The organic layer was combined and dried over Na_2_SO_4_, concentrated *in vacuo*, and then purified by flash column chromatography (petroleum ether/ethyl acetate = 4:1) to give **19c** (64 mg, 65%, over two steps) as a white solid.

After a solution of **19c** (64 mg, 0.098 mmol) was dissolved in THF (3 mL), tetrabutylammonium fluoride (1.0 M in THF, 0.5 mL, 0.53 mmol) was added, and the resulting mixture was stirred for 24 h at 60°C, and then was quenched with NH_4_Cl (aq.). After the reaction was terminated, the aqueous layer was extracted with EtOAc. The combined organic layers were washed with H_2_O and brine, dried over anhydrous Na_2_SO_4_, and concentrated *in vacuo* to produce a crude oil, which was purified by column chromatography (petroleum ether/ethyl acetate = 2:1). The crude product was directly dissolved in DCM (3 mL), and then DMP (69 mg, 0.15 mmol) and NaHCO_3_ (17 mg, 0.20 mmol) were added sequentially at 0 °C. The resultant mixture was stirred for 2 h at the same temperature. Then, the saturated aqueous Na_2_S_2_O_3_ (10 mL) was added to the mixture. The mixture was stirred for 0.5 h at room temperature and extracted with DCM (3 × 5 mL). The combined organic layers were washed with brine, then dried over Na_2_SO_4_. The solvent was removed under vacuum, and the residue was purified by column chromatography (petroleum ether/ethyl acetate = 4:1) to afford **20c** (32 mg, 60% over two steps) as a white solid. ^**1**^ **H NMR** (400 MHz, CDCl_3_) *δ* 8.62 (ddd, J = 4.9, 1.8, 1.0 Hz, 1H), 8.19 (s, 1H), 8.17–8.13 (m, 1H), 7.82 (td, J = 7.8, 1.8 Hz, 1H), 7.67 (d, J = 8.3 Hz, 1H), 7.28 (ddd, J = 7.7, 4.9, 1.2 Hz, 1H), 7.17 (ddd, J = 8.4, 7.3, 1.5 Hz, 1H), 6.94 (td, J = 7.4, 1.0 Hz, 1H), 6.86 (dd, J = 7.7, 1.4 Hz, 1H), 6.26 (d, J = 5.6 Hz, 1H), 6.01 (s, 1H), 5.51 (ddd, J = 6.2, 4.1, 2.5 Hz, 1H), 5.42 (s, 1H), 5.13 (d, J = 16.6 Hz, 1H), 5.04 (d, J = 16.6 Hz, 1H), 4.18–4.10 (m, 1H), 3.63 (ddd, J = 10.9, 9.2, 5.6 Hz, 1H), 2.99 (dd, J = 18.0, 4.1 Hz, 1H), 2.35 (dd, J = 17.9, 2.6 Hz, 1H), 2.29–2.15 (m, 2H), 2.10 (d, J = 9.9 Hz, 1H), 1.59 (s, 9H). ^**13**^ **C NMR** (101 MHz, CDCl_3_) *δ* 196.2, 164.8, 151.4, 149.7, 149.4, 149.2, 146.4, 142.7, 137.0, 129.8, 129.3, 124.1, 123.8, 123.7, 123.3, 121.6, 120.1, 115.0, 103.7, 83.1, 67.4, 60.1, 53.4, 48.3, 41.8, 38.4, 28.3. **HRMS (ESI)**: C_29_H_31_N_6_O_5_^+^ [(M + H)^+^]: calcd: 543.2350; found: 543.2352.

### 3.2 Cell culture and drug administration

MH7A cells (Jennio Biotech Co., Ltd, China) were cultured as we reported previously. (Chen et al., 2022). For cell viability detection, MH7A cells were seeded in 96-well plates overnight before being stimulated with different dosages of compounds (2.5–20 μM) for 24 h. Subsequently, the treatment of the resulting cells was co-incubated with CCK-8 solution (10 μL/well; APExBIO Corporation) for 1 h. Finally, the absorbance of each well was tested through a microplate reader (450 nm; BioTek, United States).

## 4 Conclusion

In this study, we synthesized a series of novel akuammiline alkaloid derivatives, among which **9** and **17c** exhibited the optimal inhibitory effect on the proliferation of RA-FLSs with an IC_50_ of (3.22 ± 0.29 μM) and (3.21 ± 0.31 μM), respectively. These results suggested that **9** and **17c** may have potential activity in treating RA as lead compounds.

In summary, we presented the flexible synthetic process for creating analogs of akuammiline alkaloids with various skeletons and analyzed their effects on the proliferation of RA-FLSs *in vitro* from their structure. We also demonstrated that **9** and **17c** elicited a strong inhibitory effect on the proliferation of RA-FLSs. Our study is certain to lay a good foundation for further pharmacology studies of akuammiline alkaloid derivatives and provides a good example for the development of anti-RA small molecule drugs that originate from natural products.

## Data Availability

The original contributions presented in the study are included in the article/Supplementary Material, further inquiries can be directed to the corresponding authors.
