# Supplementary material for: Akuammiline alkaloid derivatives: divergent synthesis and effect on the proliferation of rheumatoid arthritis fibroblast-like synoviocytes
Source: Front Chem. 2023 Apr 28;11:1179948. doi: 10.3389/fchem.2023.1179948 (PMC10176115; doi:10.3389/fchem.2023.1179948)
Supplement: Supplementary file 1 [file DataSheet1.pdf]

Supplementary Material

Table of Contents

1. **General Information** 3
2. **Analytical data of desired products** 4
3. **Copies of ^{1}H and ^{13}C NMR spectra of products** 13
4. **Details of Biological evaluation** 31
5. **Reference** 32

1. General Information

All reactions involving air or moisture sensitive reagents, or intermediates were carried out under an argon atmosphere with dry solvents under anhydrous conditions, unless otherwise noted. Reagents were purchased at the highest commercial quality and used without further purification, unless otherwise stated. Solvent purification was conducted according to Purification of Laboratory Chemicals (Peerrin, D. D. Armarego, W. L. and Perrins, D. R., Pergamon Press: Oxford, **1980**). Yields refer to isolated compounds, unless otherwise stated. Reactions were monitored by thin-layer chromatography (TLC) carried out on 0.25 mm Tsingdao silica gel plates (60F-254).

Visualization on TLC was achieved by use of UV light at 254 nm, exposure to iodine vapor. Staining was performed with an ethanolic solution of phosphomolybdic acid (PMA) and cerium sulfate, or by oxidative staining with an aqueous basic potassium permanganate ($KMnO_4$) solution and subsequent heating. Tsingdao silica gel (60, particle size 0.040 – 0.063 mm) was used for flash column chromatography. NMR spectra were recorded on a Brüker AVANCE 400 (^1H: 400 MHz, ^{13}C: 100 MHz) or a Brüker AVANCE 500 (^1H: 500 MHz, ^{13}C: 125 MHz) instrument. Chemical shifts were reported in parts per million (ppm) with respect to the residual solvent signal $CDCl_3$ (^1H NMR: $\delta = 7.26$; ^{13}C NMR: $\delta = 77.16$). Peak multiplicities were reported as follows: s = singlet, d = doublet, t = triplet, q = quartet, dd = doublet of doublets, td = triplet of doublets, dt = doublet of triplets, ddd = doublet of doublet of doublets, m = multiplet, br = broad signal. High resolution mass spectra (HRMS) were recorded on an Agilent Mass spectrometer using ESI-TOF (electrospray ionization-time of flight). Optical rotation values were recorded on a Rudolph Research Analytical Autopol I polarimeter (Rudolph Research Co.) The substrates were prepared according to literature methods.[1, 2]

Note: There exist rotational isomers for few compounds containing the acetal group, which are difficult to separate using column chromatography.

2. Analytical data of desired products

Compound 9

tert-butyl(4bS,8S,8aR)-8-azido-5-methylene-6-oxo-5,6,7,8-tetrahydro-9H-8a,4b-(epoxyethano) carbazole-9-carboxylate

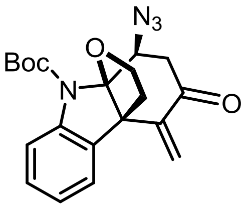

A white solid, 91 mg, 85% yield.
TLC: R_f = 0.45 (Petroleum ether/EtOAc = 4:1) [UV].
^{1}H NMR (400 MHz, $CDCl_3$) δ 7.72 – 7.65 (m, 1H), 7.20 (ddd, J = 8.5, 6.2, 2.6 Hz, 1H), 7.05 – 6.95 (m, 2H), 6.21 (s, 1H), 5.64 (s, 1H), 5.34 – 5.29 (m, 1H), 4.32 (ddd, J = 8.6, 7.4, 1.1 Hz, 1H), 3.76 (ddd, J = 11.9, 9.1, 4.9 Hz, 1H), 2.66 – 2.51 (m, 2H), 2.48 (d, J = 4.7 Hz, 1H), 2.35 (dd, J = 17.4, 2.4 Hz, 1H), 1.62 (s, 9H).
^{13}C NMR (101 MHz, $CDCl_3$) δ 195.5, 151.8, 147.0, 142.4, 130.5, 129.2, 124.2, 123.9, 120.5, 115.0, 105.3, 83.0, 67.9, 60.0, 58.0, 42.3, 39.2, 28.3.
HRMS (ESI): $C_{20}H_{22}N_4NaO_4^+$ [$(M+Na)^+$]: calcd: 405.1533; found: 405.1531.

Compound 10

(4bS,8S,8aR)-8-azido-5-methylene-7,8-dihydro-9H-8a,4b-(epoxyethano) carbazol-6(5H)-one

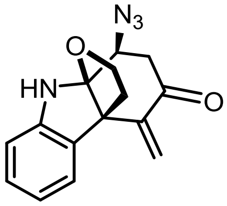

A white solid, 26.8 mg, 40% yield.
TLC: R_f = 0.38 (Petroleum ether/EtOAc = 2:1) [UV].
^{1}H NMR (400 MHz, $CDCl_3$) δ 7.09 (td, J = 7.6, 1.3 Hz, 1H), 7.01 (dd, J = 7.6, 1.3 Hz, 1H), 6.80 (td, J = 7.5, 1.0 Hz, 1H), 6.61 (d, J = 7.8 Hz, 1H), 6.23 (d, J = 0.8 Hz, 1H), 5.68 (s, 1H), 4.42 (s, 1H), 4.29 (ddd, J = 8.8, 4.9, 3.0 Hz, 1H), 4.17 (dd, J = 4.9, 2.8 Hz, 1H), 3.82 – 3.68 (m, 1H), 2.77 (dd, J = 17.3, 2.9 Hz, 1H), 2.62 (dd, J = 17.3, 4.9 Hz, 1H), 2.55 – 2.48 (m, 2H).
^{13}C NMR (101 MHz, $CDCl_3$) δ 196.4, 148.0, 147.0, 129.8, 129.1, 124.2, 121.6, 120.3, 108.8, 103.1, 68.0, 60.5, 60.1, 43.0, 40.1.

HRMS (ESI): $C_{15}H_{15}N_4O_2^+$ [(M+H)$^+$]: calcd: 283.1190; found: 283.1189.

Compound 13

9-(*tert*-butyl) 5-methyl (4bS,8S,8aR)-8-azido-7,8-dihydro-9H-8a,4b-(epoxyethano) carbazole-5,9-dicarboxylate

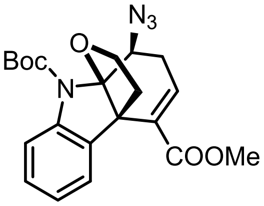

A white solid, 55 mg, 78% yield.
TLC: R_f = 0.62 (Petroleum ether/EtOAc = 5:1) [UV].
^{1}H NMR (400 MHz, $CDCl_3$) δ 7.70 (d, J = 8.3 Hz, 1H), 7.61 (ddd, J = 7.7, 1.5, 0.6 Hz, 1H), 7.20 (ddd, J = 8.2, 7.4, 1.5 Hz, 1H), 6.98 (td, J = 7.5, 1.1 Hz, 1H), 6.87 (td, J = 4.7, 0.8 Hz, 1H), 5.46 (s, 1H), 3.69 (ddd, J = 12.3, 8.6, 4.9 Hz, 1H), 3.07 (dd, J = 12.6, 4.9 Hz, 1H), 2.56 (td, J = 12.5, 7.9 Hz, 1H), 2.43 (dd, J = 4.6, 3.1 Hz, 2H), 1.63 (s, 9H).
^{13}C NMR (101 MHz, $CDCl_3$) δ 165.7, 152.3, 142.2, 135.4, 134.5, 132.0, 128.7, 124.9, 123.4, 115.4, 105.3, 82.9, 68.8, 57.8, 57.6, 51.2, 39.3, 28.6, 28.4.
HRMS (ESI): $C_{21}H_{24}N_4NaO_5^+$ [(M+Na)$^+$]: calcd: 435.1639; found: 435.1642.

Compound 5

***tert*-butyl (4bS,6S,8S,8aR)-8-azido-6-hydroxy-11-methoxy-5-methylene-5,6,7,8-tetrahydro-9H-8a,4b-(epoxyethano) carbazole-9-carboxylate**

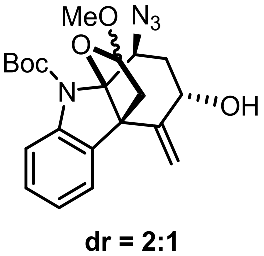

dr = 2:1

A white solid, 68 mg, 88% yield.
TLC: R_f = 0.63 (Petroleum ether/EtOAc = 2:1) [UV].
^{1}H NMR (400 MHz, $CDCl_3$) δ 7.75 (d, J = 8.2 Hz, 1H), 7.68 (s, 1H), 7.24 – 7.15 (m, 2H), 7.14 – 7.06 (m, 2H), 7.00 (tdd, J = 7.5, 4.3, 1.0 Hz, 2H), 5.44 – 5.32 (m, 4H), 5.30 (d, J = 5.1 Hz, 1H), 5.13 (s, 1H), 5.06 (dd, J = 6.7, 5.0 Hz, 1H), 4.99 (t, J = 3.7 Hz, 1H), 4.43 (t, J = 7.5 Hz, 1H), 4.37 (t, J = 7.7 Hz, 1H), 3.50 (s, 3H), 3.19 (s, 2H), 2.65 (dd, J = 12.7, 5.2 Hz, 1H), 2.60 – 2.46 (m, 3H), 2.46 – 2.30 (m, 2H), 1.63 (s, 3H), 1.61 (s, 6H), 0.97 (s, 1H), 0.82 (s, 1H).

^{13}C NMR (101 MHz, $CDCl_3$) δ 152.2, 152.0, 151.8, 150.3, 141.6, 134.1, 133.4, 128.9, 128.6, 123.4, 123.2, 122.9, 115.7, 115.6, 115.4, 114.9, 106.1, 105.7, 105.6, 104.6, 82.4, 82.3, 68.5, 68.1, 58.9, 57.9, 57.4, 54.9, 46.9, 45.4, 33.1, 32.5, 28.4.
HRMS (ESI): $C_{21}H_{26}N_4NaO_5^+$ [(M+Na)$^+$]: calcd: 437.1795; found: 437.1782.

Compound 6
tert-butyl (4bS,8S,8aR)-8-azido-11-methoxy-5-methylene-6-oxo-5,6,7,8-tetrahydro-9H-8a,4b-(epoxyethano) carbazole-9-carboxylate

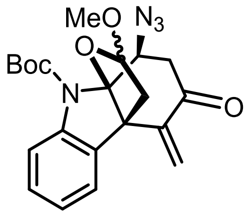

dr = 2:1

A white solid, 43 mg, 86% yield.
TLC: R_f = 0.49 (Petroleum ether/EtOAc = 4:1) [UV].
^{1}H NMR (400 MHz, $CDCl_3$) δ 7.66 (s, 1H), 7.18 (ddd, J = 8.5, 4.9, 3.8 Hz, 1H), 6.99 (dd, J = 4.0, 0.9 Hz, 2H), 6.18 (s, 1H), 5.58 (s, 1H), 5.42 (dd, J = 3.3, 2.2 Hz, 1H), 5.37 (s, 1H), 3.23 (s, 3H), 2.72 – 2.66 (m, 2H), 2.58 (dd, J = 17.6, 4.3 Hz, 1H), 2.40 (dd, J = 17.6, 2.4 Hz, 1H), 1.65 (s, 9H).
^{13}C NMR (101 MHz, $CDCl_3$) δ 195.2, 151.7, 147.3, 132.0, 128.9, 123.9, 123.8, 120.3, 115.4, 106.8, 105.4, 82.8, 55.1, 48.1, 39.0, 28.4.
HRMS (ESI): $C_{21}H_{24}N_4NaO_5^+$ [(M+Na)$^+$]: calcd: 435.1639; found: 435.1640.

Compound 8(up)
9-(_tert_-butyl) 5-methyl (4bS,8S,8aR)-8-azido-11-methoxy-7,8-dihydro-9H-8a,4b-(epoxyethano) carbazole-5,9-dicarboxylate

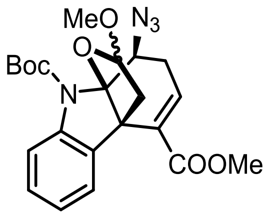

dr = 2:1

A white solid, 67 mg, 59% yield.
TLC: R_f = 0.58 (Petroleum ether/EtOAc = 7:1) [UV].
^{1}H NMR (400 MHz, $CDCl_3$) δ 7.72 (d, J = 8.2 Hz, 1H), 7.61 (dd, J = 7.7, 1.4 Hz, 1H), 7.21 (ddd, J = 8.3, 7.4, 1.4 Hz, 1H), 6.99 (td, J = 7.5, 1.1 Hz, 1H), 6.86 – 6.79 (m, 1H), 5.42 (s, 1H), 5.06 (dd, J = 7.8, 4.9 Hz, 1H), 3.74 (s, 3H), 3.49 (s, 3H), 3.34 (dd, J = 13.4, 4.9 Hz, 1H), 2.54 – 2.44 (m, 3H), 1.62 (s, 9H).

^{13}C NMR (101 MHz, $CDCl_3$) δ 165.5, 152.3, 141.3, 134.9, 133.9, 132.3, 128.9, 125.1, 123.5, 116.0, 107.2, 104.0, 82.9, 58.6, 57.4, 55.9, 51.8, 44.1, 29.2, 28.4.
HRMS (ESI): $C_{22}H_{26}N_4NaO_6^+$ [(M+Na)$^+$]: calcd: 465.1745; found: 465.1743.

Compound 8(down)
9-(*tert*-butyl) 5-methyl
(4bS,8S,8aR)-8-azido-11-methoxy-7,8-dihydro-9H-8a,4b-(epoxyethano) carbazole-5,9-dicarboxylate

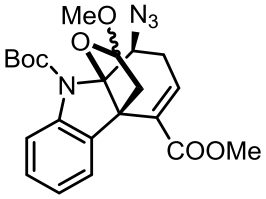

dr = 2:1

A white solid, 67 mg, 59% yield.
TLC: R_f = 0.56 (Petroleum ether/EtOAc = 7:1) [UV].
^{1}H NMR (400 MHz, $CDCl_3$) δ 7.70 (d, J = 8.0 Hz, 1H), 7.64 – 7.51 (m, 1H), 7.25 – 7.12 (m, 1H), 6.97 (td, J = 7.5, 1.1 Hz, 1H), 6.84 (dt, J = 9.8, 4.6 Hz, 1H), 5.43 (t, J = 3.1 Hz, 1H), 5.30 (d, J = 5.1 Hz, 1H), 3.76 (d, J = 13.9 Hz, 3H), 3.59 – 3.46 (m, 1H), 3.17 (d, J = 13.6 Hz, 1H), 3.10 (s, 2H), 2.58 (dd, J = 13.6, 5.2 Hz, 1H), 2.53 – 2.38 (m, 2H), 1.63 (d, J = 6.2 Hz, 9H).
^{13}C NMR (101 MHz, $CDCl_3$) δ 165.6, 152.2, 140.6, 135.2, 134.9, 134.3, 128.1, 124.1, 123.5, 115.6, 106.1, 105.8, 82.6, 58.2, 55.9, 54.6, 51.8, 45.8, 28.4, 28.0.
HRMS (ESI): $C_{22}H_{26}N_4NaO_6^+$ [(M+Na)$^+$]: calcd: 465.1745; found: 465.1739.

Compound 17a
***tert*-butyl**
(4bS,8S,8aR)-8-acetamido-5-methylene-6-oxo-5,6,7,8-tetrahydro-9H-8a,4b-(epoxyethano) carbazole-9-carboxylate

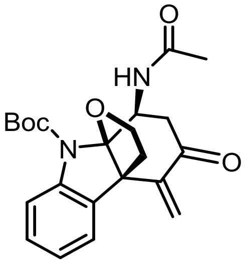

A white solid, 38 mg, 72% yield.
TLC: R_f = 0.42 (Petroleum ether/EtOAc = 4:1) [UV].
^{1}H NMR (400 MHz, $CDCl_3$) δ 7.70 (d, J = 8.3 Hz, 1H), 7.20 (ddd, J = 8.4, 6.5, 2.3 Hz, 1H), 7.02 – 6.92 (m, 2H), 6.29 (s, 1H), 5.67 (d, J = 5.8 Hz, 2H), 5.39 (q, J = 2.0 Hz, 1H), 4.30 – 4.21 (m, 1H), 3.75 (ddd, J = 11.8, 9.4, 5.2 Hz, 1H), 3.07 (dd, J = 17.6, 4.4 Hz, 1H), 2.53 – 2.35 (m, 2H), 2.32 (dd, J = 17.6, 2.6 Hz, 1H), 1.93 (s, 3H), 1.59

(s, 9H).
^{13}C NMR (101 MHz, $CDCl_3$) δ 196.5, 170.2, 151.4, 146.6, 142.8, 130.1, 129.3, 124.1, 123.7, 121.3, 115.0, 104.1, 82.9, 67.2, 60.2, 48.6, 42.4, 38.9, 28.3, 23.5.
HRMS (ESI): $C_{22}H_{26}N_2NaO_5^+$ [$(M+Na)^+$]: calcd: 421.1734; found: 421.1737.

Compound 17b

***tert*-butyl (4bS,8S,8aR)-5-methylene-6-oxo-8-(2,2,2-trifluoroacetamido)-5,6,7,8-tetrahydro-9H-8a,4b-(epoxyethano) carbazole-9-carboxylate**

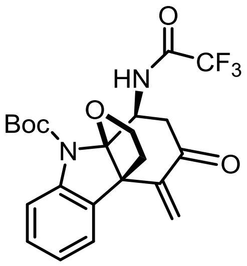

A white solid, 44 mg, 69% yield.
TLC: R_f = 0.39 (Petroleum ether/EtOAc = 4:1) [UV].
^{1}H NMR (400 MHz, $CDCl_3$) δ 7.68 (d, J = 8.2 Hz, 1H), 7.22 (ddd, J = 8.5, 6.5, 2.3 Hz, 1H), 7.05 – 6.94 (m, 2H), 6.70 (s, 1H), 6.29 (s, 1H), 5.71 (s, 1H), 5.40 (td, J = 4.9, 2.9 Hz, 1H), 4.35 – 4.24 (m, 1H), 3.84 – 3.72 (m, 1H), 3.08 (dd, J = 17.9, 4.7 Hz, 1H), 2.51 – 2.39 (m, 3H), 1.59 (s, 9H).
^{13}C NMR (101 MHz, $CDCl_3$) δ 195.3, 157.5, 157.1, 156.8, 156.4, 151.4, 146.1, 142.4, 129.6, 129.4, 124.1, 124.0, 122.3, 119.7, 116.8, 115.1, 113.9, 111.1, 103.4, 83.4, 67.5, 60.3, 49.2, 42.3, 37.9, 28.2.
^{19}F NMR (376 MHz, $CDCl_3$) δ -76.0.
HRMS (ESI): $C_{22}H_{23}F_3N_2NaO_5^+$ [$(M+Na)^+$]: calcd: 475.1451; found: 475.1452.

Compound 17c

***tert*-butyl (4bS,8S,8aR)-5-methylene-8-((4-methylphenyl) sulfonamido)-6-oxo-5,6,7,8-tetrahydro-9H-8a,4b-(epoxyethano) carbazole-9-carboxylate**

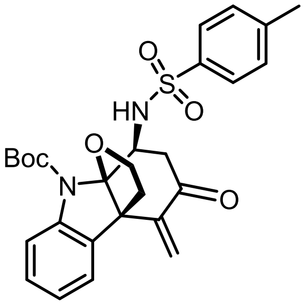

A white solid, 84 mg, 78% yield.
TLC: R_f = 0.48 (Petroleum ether/EtOAc = 4:1) [UV].
^{1}H NMR (400 MHz, $CDCl_3$) δ 7.74 (d, J = 8.0 Hz, 2H), 7.65 (d, J = 8.2 Hz, 1H), 7.31

(d, J = 8.0 Hz, 2H), 7.16 (ddd, J = 8.5, 6.5, 2.3 Hz, 1H), 7.00 – 6.90 (m, 2H), 6.15 (s, 1H), 5.56 (s, 1H), 5.24 (s, 1H), 4.74 (dt, J = 3.9, 1.8 Hz, 1H), 4.16 (dd, J = 9.2, 7.3 Hz, 1H), 3.65 (ddd, J = 12.1, 9.2, 4.8 Hz, 1H), 2.95 (dd, J = 17.9, 4.3 Hz, 1H), 2.49 (dt, J = 12.1, 6.0 Hz, 1H), 2.43 (s, 3H), 2.33 (dd, J = 11.9, 4.7 Hz, 1H), 2.25 (dt, J = 17.9, 1.7 Hz, 1H), 1.41 (s, 9H).
^{13}C NMR (101 MHz, $CDCl_3$) δ 195.5, 151.4, 146.6, 143.8, 142.6, 136.2, 130.2, 129.7, 129.1, 127.3, 124.2, 123.9, 120.2, 114.9, 103.5, 82.7, 67.6, 60.2, 51.1, 42.0, 38.2, 28.0, 21.5.
HRMS (ESI): $C_{27}H_{30}N_2NaO_6S^+$ [$(M+H)^+$]: calcd: 533.1717; found: 533.1720.

Compound 17d
<u>*tert*-butyl (4bS,8S,8aR)-8-((2,4-dichlorophenyl) sulfonamido)-5-methylene-6-oxo-5,6,7,8-tetrahydro-9H-8a,4b-(epoxyethano) carbazole-9-carboxylate</u>

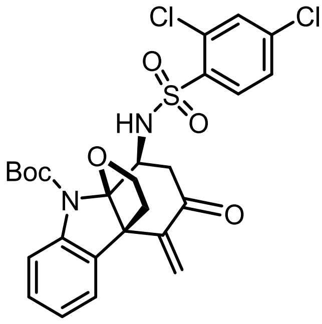

A white solid, 63 mg, 74% yield.
TLC: R_f = 0.45 (Petroleum ether/EtOAc = 4:1) [UV].
^{1}H NMR (400 MHz, $CDCl_3$) δ 8.01 (d, J = 8.5 Hz, 1H), 7.65 (d, J = 8.3 Hz, 1H), 7.53 (d, J = 2.0 Hz, 1H), 7.42 (dd, J = 8.5, 2.0 Hz, 1H), 7.17 (ddd, J = 8.5, 6.9, 1.9 Hz, 1H), 7.02 – 6.90 (m, 2H), 6.17 – 6.12 (m, 1H), 5.63 (s, 1H), 5.50 (d, J = 2.7 Hz, 1H), 4.97 – 4.90 (m, 1H), 4.10 (dd, J = 9.3, 7.3 Hz, 1H), 3.67 (ddd, J = 12.2, 9.3, 4.8 Hz, 1H), 2.85 (dd, J = 18.0, 4.1 Hz, 1H), 2.50 (td, J = 12.0, 7.4 Hz, 1H), 2.38 (dd, J = 11.9, 4.7 Hz, 1H), 2.34 – 2.24 (m, 1H), 1.49 (s, 9H).
^{13}C NMR (101 MHz, $CDCl_3$) δ 195.4, 151.3, 146.4, 142.6, 139.7, 135.9, 132.8, 132.0, 131.3, 129.9, 129.3, 127.5, 124.2, 124.0, 120.9, 115.1, 103.4, 83.0, 67.7, 60.2, 51.5, 42.1, 38.7, 28.2.
HRMS (ESI): $C_{26}H_{26}Cl_2N_2NaO_6S^+$ [$(M+Na)^+$]: calcd: 587.0781; found: 587.0781.

Compound 17e
<u>*tert*-butyl (4bS,8S,8aR)-8-((4-methoxyphenyl) sulfonamido)-5-methylene-6-oxo-5,6,7,8-tetrahydro-9H-8a,4b-(epoxyethano) carbazole-9-carboxylate</u>

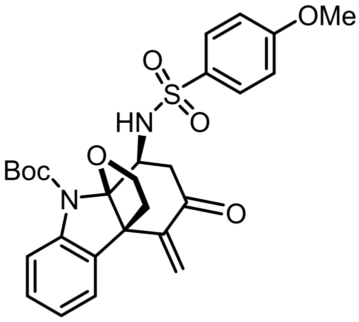

A white solid, 54 mg, 70% yield.
TLC: R_f = 0.47 (Petroleum ether/EtOAc = 4:1) [UV].
^{1}H NMR (400 MHz, $CDCl_3$) δ 7.83 – 7.74 (m, 2H), 7.66 (d, J = 8.2 Hz, 1H), 7.17 (ddd, J = 8.5, 6.6, 2.2 Hz, 1H), 7.02 – 6.91 (m, 4H), 6.16 (s, 0H), 5.57 (s, 1H), 5.11 (s, 1H), 4.77 (dt, J = 3.8, 1.7 Hz, 1H), 4.19 (dd, J = 9.3, 7.3 Hz, 1H), 3.88 (s, 3H), 3.68 (ddd, J = 12.2, 9.3, 4.8 Hz, 1H), 2.94 (dd, J = 17.9, 4.2 Hz, 1H), 2.49 (td, J = 12.1, 7.4 Hz, 1H), 2.36 (dd, J = 11.9, 4.7 Hz, 1H), 2.25 (ddd, J = 17.8, 2.1, 1.2 Hz, 1H), 1.45 (s, 9H).
^{13}C NMR (101 MHz, $CDCl_3$) δ 195.4, 163.2, 151.4, 146.7, 142.6, 130.8, 130.2, 129.5, 129.2, 124.2, 124.0, 120.2, 115.0, 114.4, 103.5, 82.8, 67.6, 60.2, 55.6, 51.2, 42.2, 38.1, 28.1.
HRMS (ESI): $C_{27}H_{30}N_2NaO_7S^+$ [(M+Na)$^+$]: calcd: 549.1666; found: 549.1669.

Compound 17f
<u>*tert*-butyl (4bS,8S,8aR)-5-methylene-8-((4-nitrophenyl) sulfonamido)-6-oxo-5,6,7,8-tetrahydro-9H-8a,4b-(epoxyethano) carbazole-9-carboxylate</u>

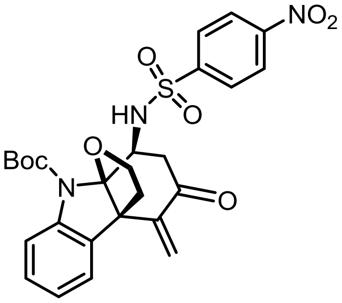

A brown solid, 58 mg, 73% yield.
TLC: R_f = 0.36 (Petroleum ether/EtOAc = 4:1) [UV].
^{1}H NMR (400 MHz, $CDCl_3$) δ 8.38 (d, J = 8.9 Hz, 2H), 8.06 (d, J = 8.9 Hz, 2H), 7.60 (d, J = 8.3 Hz, 1H), 7.19 (ddd, J = 8.5, 7.1, 1.7 Hz, 1H), 7.03 – 6.91 (m, 2H), 6.18 (s, 1H), 5.63 (s, 1H), 5.38 – 5.33 (m, 1H), 5.00 (dt, J = 4.4, 2.2 Hz, 1H), 4.20 – 4.11 (m, 1H), 3.69 (ddd, J = 12.0, 9.3, 5.0 Hz, 1H), 2.90 (dd, J = 17.8, 4.2 Hz, 1H), 2.47 (td, J = 12.0, 7.4 Hz, 1H), 2.40 – 2.35 (m, 1H), 2.32 (ddd, J = 17.8, 2.3, 1.0 Hz, 1H), 1.52 (s, 9H).
^{13}C NMR (101 MHz, $CDCl_3$) δ 195.0, 151.2, 150.2, 146.2, 145.2, 142.3, 129.9, 129.4, 128.7, 124.3, 124.2, 124.1, 121.3, 115.0, 103.3, 83.3, 67.6, 60.1, 51.3, 42.3,

38.8, 28.2.
HRMS (ESI): $C_{26}H_{27}N_3NaO_8S^+$ [$(M+Na)^+$]: calcd: 567.1411; found: 567.1412.

Compound 20a
***tert*-butyl (4bS,8S,8aR)-8-(2-(4-(4-methoxyphenyl)-1H-1,2,3-triazol-1-yl) acetamido)-5-methylene-6-oxo-5,6,7,8-tetrahydro-9H-8a,4b-(epoxyethano) carbazole-9-carboxylate**

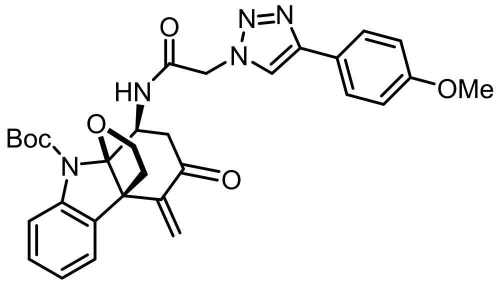

A white solid, 39 mg, 65% yield.
TLC: R_f = 0.55 (Petroleum ether/EtOAc = 3:1) [UV].
^{1}H NMR (400 MHz, $CDCl_3$) δ 7.81 – 7.72 (m, 3H), 7.66 (d, J = 8.3 Hz, 1H), 7.17 (ddd, J = 8.4, 7.3, 1.4 Hz, 1H), 7.04 – 6.98 (m, 2H), 6.94 (td, J = 7.5, 1.0 Hz, 1H), 6.86 (dd, J = 7.7, 1.5 Hz, 1H), 6.23 (d, J = 5.4 Hz, 1H), 5.97 (s, 1H), 5.53 – 5.45 (m, 1H), 5.40 (s, 1H), 5.12 (d, J = 16.5 Hz, 1H), 4.98 (d, J = 16.5 Hz, 1H), 4.14 (dd, J = 9.0, 7.3 Hz, 1H), 3.87 (s, 3H), 3.63 (ddd, J = 11.8, 9.2, 5.0 Hz, 1H), 3.01 (dd, J = 17.8, 4.1 Hz, 1H), 2.34 (dd, J = 17.8, 2.5 Hz, 1H), 2.28 – 2.13 (m, 2H), 1.60 (s, 9H).
^{13}C NMR (101 MHz, $CDCl_3$) δ 196.2, 165.2, 160.0, 151.4, 148.6, 146.3, 142.7, 129.9, 129.3, 127.0, 124.1, 123.9, 122.4, 121.7, 120.3, 115.0, 114.5, 103.6, 83.1, 67.4, 60.1, 55.3, 53.4, 48.3, 41.9, 38.4, 29.7, 28.3.
HRMS (ESI): $C_{31}H_{34}N_5O_6^+$ [$(M+H)^+$]: calcd: 572.2504; found: 572.2505.

Compound 20b
***tert*-butyl (4bS,8S,8aR)-8-(2-(4-(4-fluorophenyl)-1H-1,2,3-triazol-1-yl) acetamido)-5-methylene-6-oxo-5,6,7,8-tetrahydro-9H-8a,4b-(epoxyethano) carbazole-9-carboxylate**

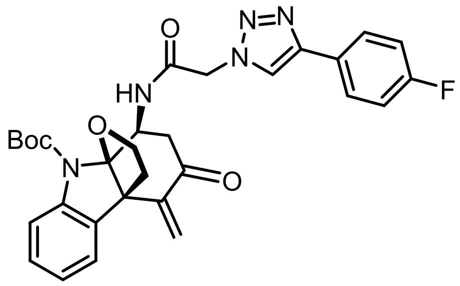

A white solid, 46 mg, 71% yield.
TLC: R_f = 0.51 (Petroleum ether/EtOAc = 3:1) [UV].
^{1}H NMR (400 MHz, $CDCl_3$) δ 7.87 – 7.76 (m, 3H), 7.64 (d, J = 8.3 Hz, 1H), 7.16 (dtd, J = 8.8, 7.2, 1.8 Hz, 3H), 6.94 (td, J = 7.5, 1.0 Hz, 1H), 6.86 (dd, J = 7.7, 1.4 Hz, 1H), 6.30 (d, J = 5.3 Hz, 1H), 5.97 (s, 1H), 5.48 (td, J = 4.5, 2.4 Hz, 1H), 5.41 (s, 1H),

5.11 (d, J = 16.4 Hz, 1H), 4.99 (d, J = 16.4 Hz, 1H), 4.17 – 4.06 (m, 1H), 3.63 (ddd, J = 11.7, 9.2, 5.1 Hz, 1H), 3.01 (dd, J = 17.7, 4.2 Hz, 1H), 2.33 (dd, J = 17.8, 2.5 Hz, 2H), 2.29 – 2.15 (m, 1H), 1.58 (s, 9H).
^{13}C NMR (101 MHz, $CDCl_3$) δ 196.1, 165.0, 164.1, 161.6, 151.4, 147.7, 146.3, 142.6, 129.8, 129.3, 127.5, 127.4, 126.1, 126.0, 124.1, 123.9, 121.6, 121.0, 116.2, 116.0, 115.0, 103.6, 83.1, 67.4, 60.0, 53.4, 48.4, 42.0, 38.4, 28.3.
^{19}F NMR (376 MHz, CDCl3) δ -112.5.
HRMS (ESI): $C_{30}H_{31}FN_5O_5^+$ [$(M+H)^+$]: calcd: 560.2304; found: 560.2308.

Compound 20c

***tert*-butyl (4bS,8S,8aR)-5-methylene-6-oxo-8-(2-(4-(pyridin-2-yl)-1H-1,2,3-triazol-1-yl) acetamido)-5,6,7,8-tetrahydro-9H-8a,4b-(epoxyethano) carbazole-9-carboxylate**

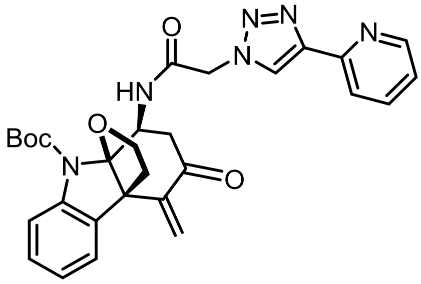

A white solid, 32 mg, 60% yield.
TLC: R_f = 0.68 (Petroleum ether/EtOAc = 2:1) [UV].
^{1}H NMR (400 MHz, $CDCl_3$) δ 8.62 (ddd, J = 4.9, 1.8, 1.0 Hz, 1H), 8.19 (s, 1H), 8.17 – 8.13 (m, 1H), 7.82 (td, J = 7.8, 1.8 Hz, 1H), 7.67 (d, J = 8.3 Hz, 1H), 7.28 (ddd, J = 7.7, 4.9, 1.2 Hz, 1H), 7.17 (ddd, J = 8.4, 7.3, 1.5 Hz, 1H), 6.94 (td, J = 7.4, 1.0 Hz, 1H), 6.86 (dd, J = 7.7, 1.4 Hz, 1H), 6.26 (d, J = 5.6 Hz, 1H), 6.01 (s, 1H), 5.51 (ddd, J = 6.2, 4.1, 2.5 Hz, 1H), 5.42 (s, 1H), 5.13 (d, J = 16.6 Hz, 1H), 5.04 (d, J = 16.6 Hz, 1H), 4.18 – 4.10 (m, 1H), 3.63 (ddd, J = 10.9, 9.2, 5.6 Hz, 1H), 2.99 (dd, J = 18.0, 4.1 Hz, 1H), 2.35 (dd, J = 17.9, 2.6 Hz, 1H), 2.29 – 2.15 (m, 2H), 2.10 (d, J = 9.9 Hz, 1H), 1.59 (s, 9H).
^{13}C NMR (101 MHz, $CDCl_3$) δ 196.2, 164.8, 151.4, 149.7, 149.4, 149.2, 146.4, 142.7, 137.0, 129.8, 129.3, 124.1, 123.8, 123.7, 123.3, 121.6, 120.1, 115.0, 103.7, 83.1, 67.4, 60.1, 53.4, 48.3, 41.8, 38.4, 28.3.
HRMS (ESI): $C_{29}H_{31}N_6O_5^+$ [$(M+H)^+$]: calcd: 543.2350; found: 543.2352.

3. Copies of ^{1}H and ^{13}C NMR spectra of products

Compound **9**

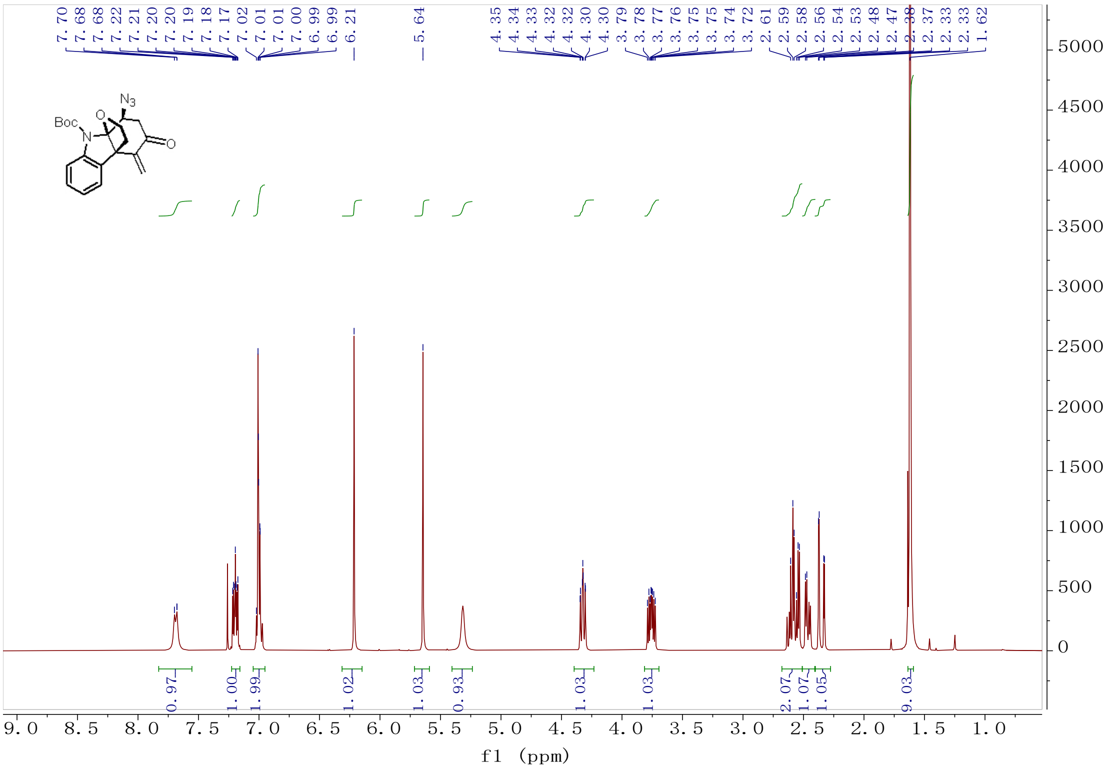

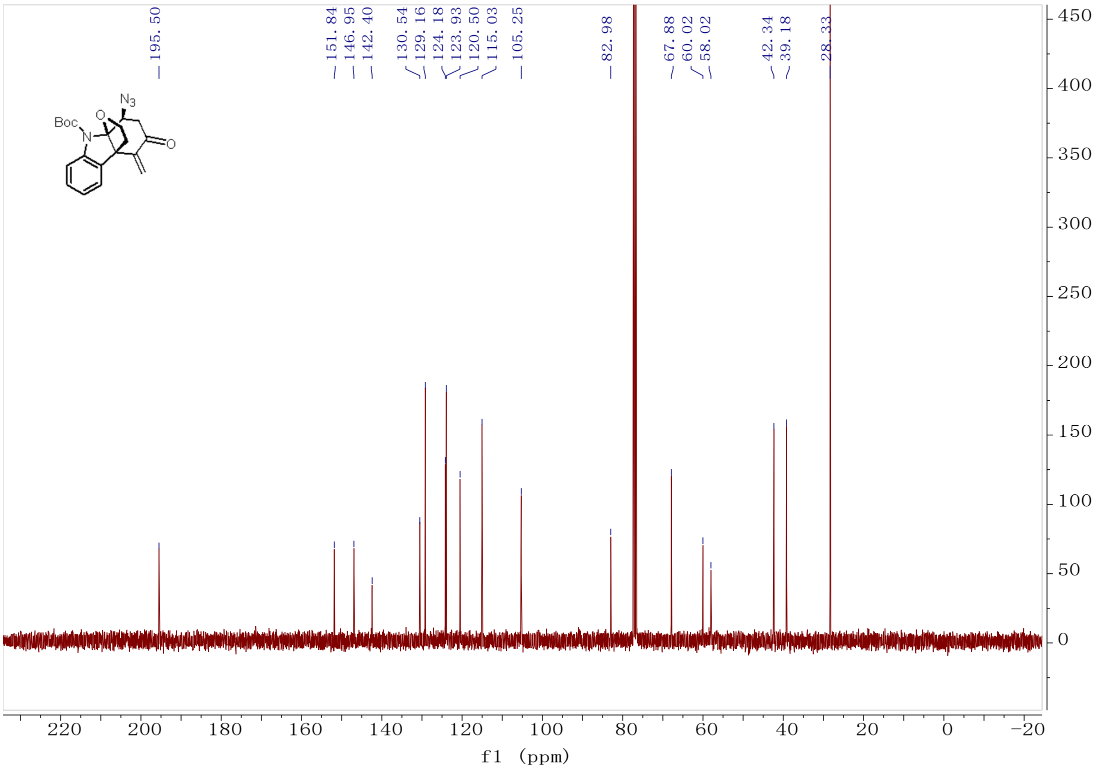

Compound **10**

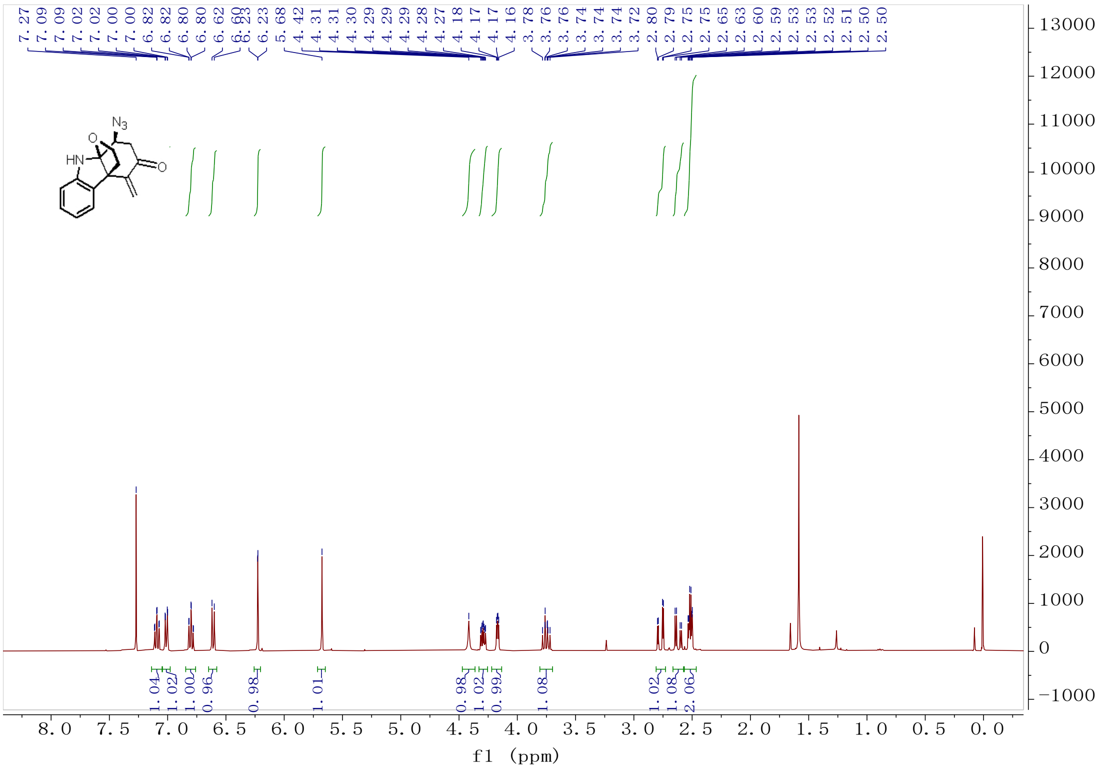
7.27
7.09
7.09
7.02
7.02
7.00
7.00
6.82
6.82
6.80
6.80
6.62
6.60
6.23
6.23
5.68
4.42
4.31
4.31
4.30
4.29
4.29
4.28
4.27
4.18
4.17
4.17
4.16
3.78
3.76
3.76
3.74
3.74
3.72
2.80
2.79
2.75
2.75
2.65
2.63
2.60
2.59
2.53
2.53
2.52
2.51
2.50
2.50
N_3
O
HN
O
1.04
1.02
1.00
0.96
0.98
1.01
0.98
1.02
0.99
1.08
1.02
1.08
2.06
f1 (ppm)

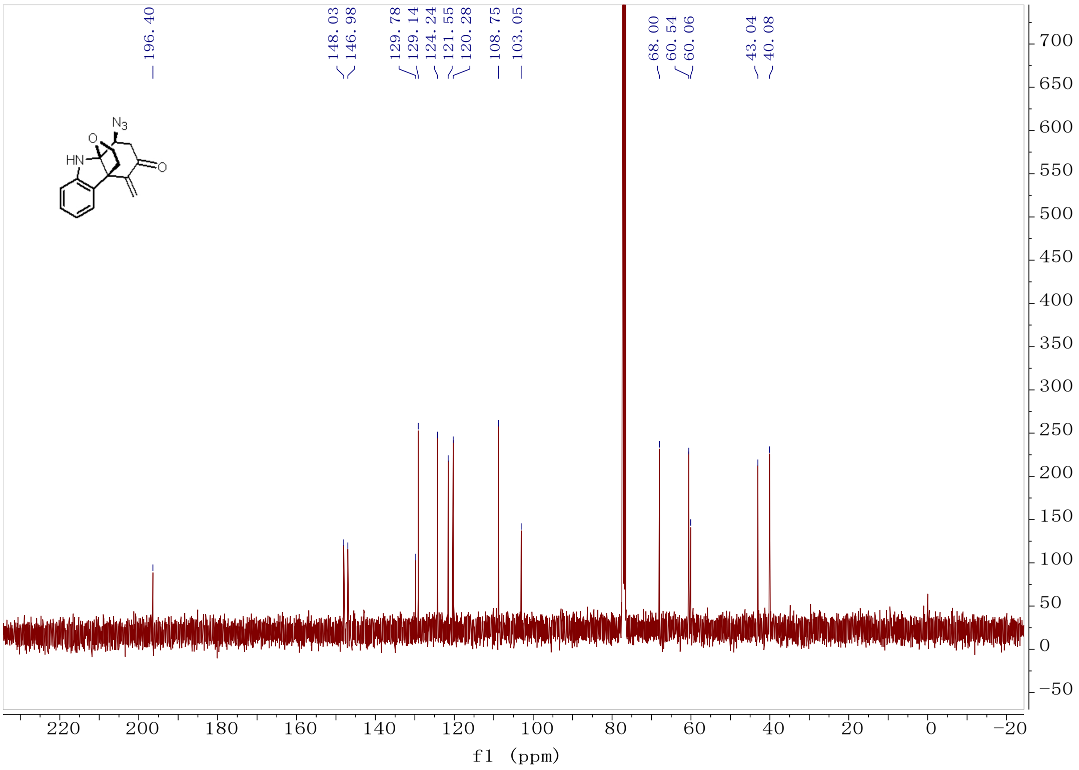
196.40
148.03
146.98
129.78
129.14
124.24
121.55
120.28
108.75
103.05
68.00
60.54
60.06
43.04
40.08
N_3
O
HN
O
f1 (ppm)

Compound **13**

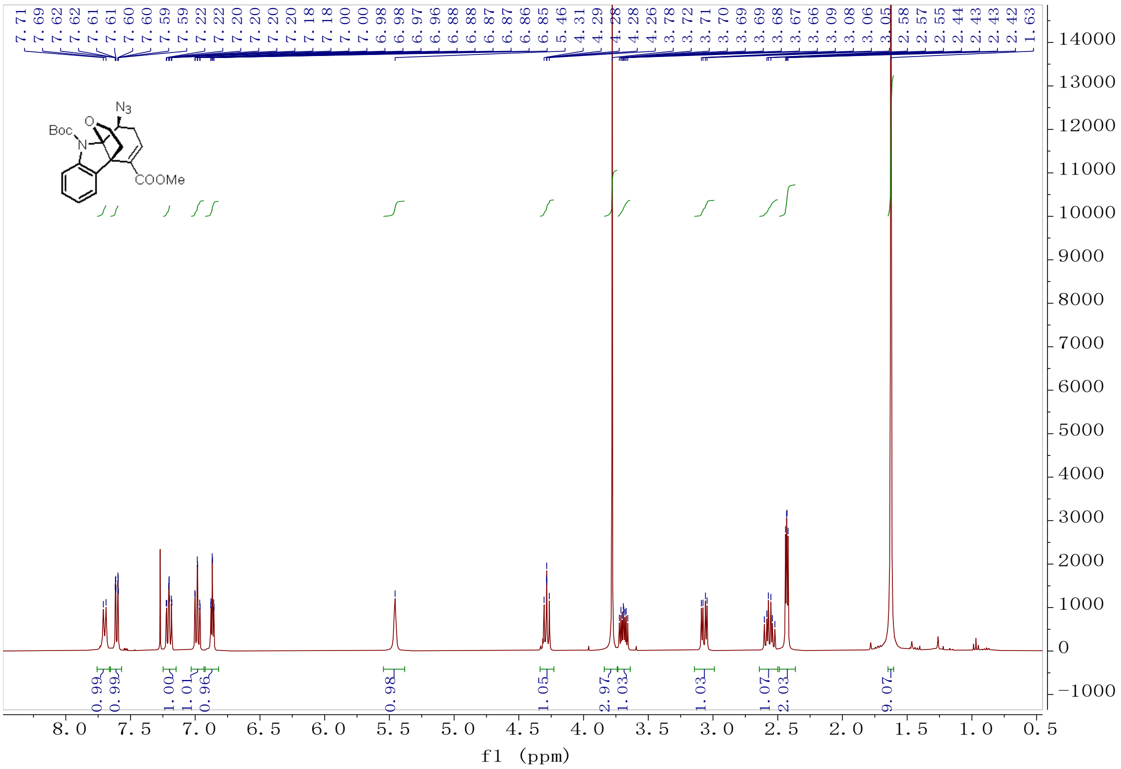
N3
Boc
O
N
COOMe
f1 (ppm)

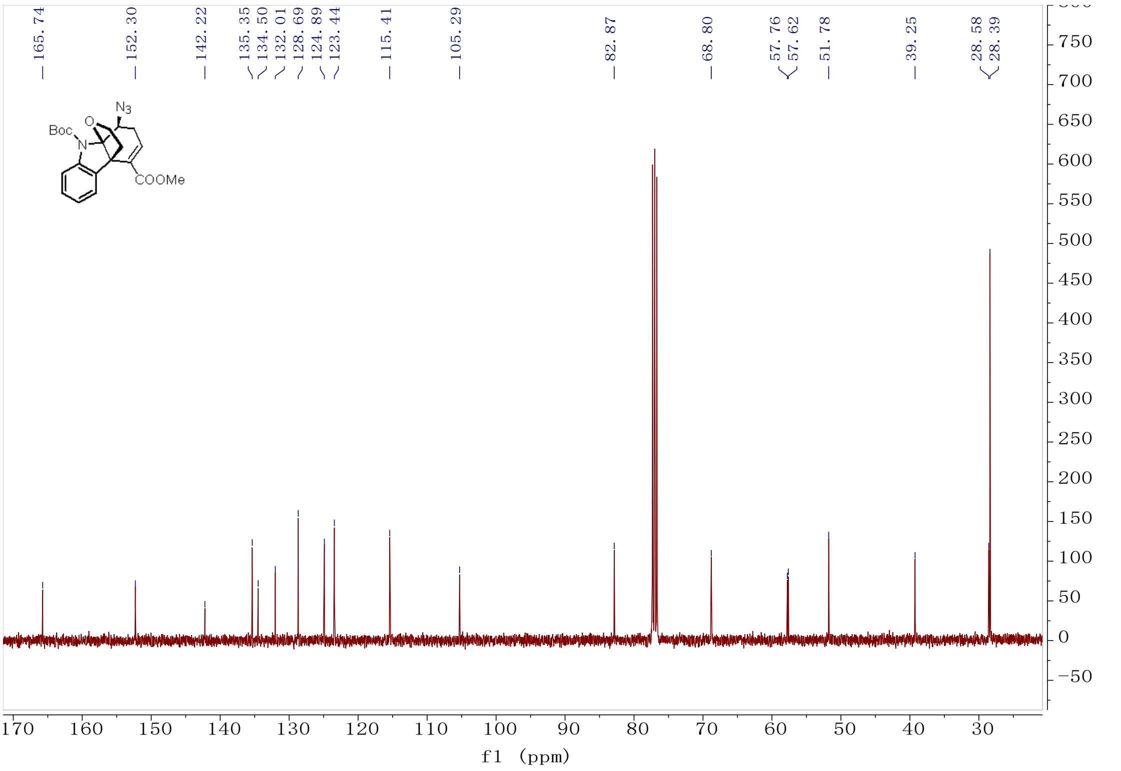
165.74
152.30
142.22
135.35
134.50
132.01
128.69
124.89
123.44
115.41
105.29
82.87
68.80
57.76
57.62
51.78
39.25
28.58
28.39
N3
Boc
O
N
COOMe
f1 (ppm)

Compound **5**

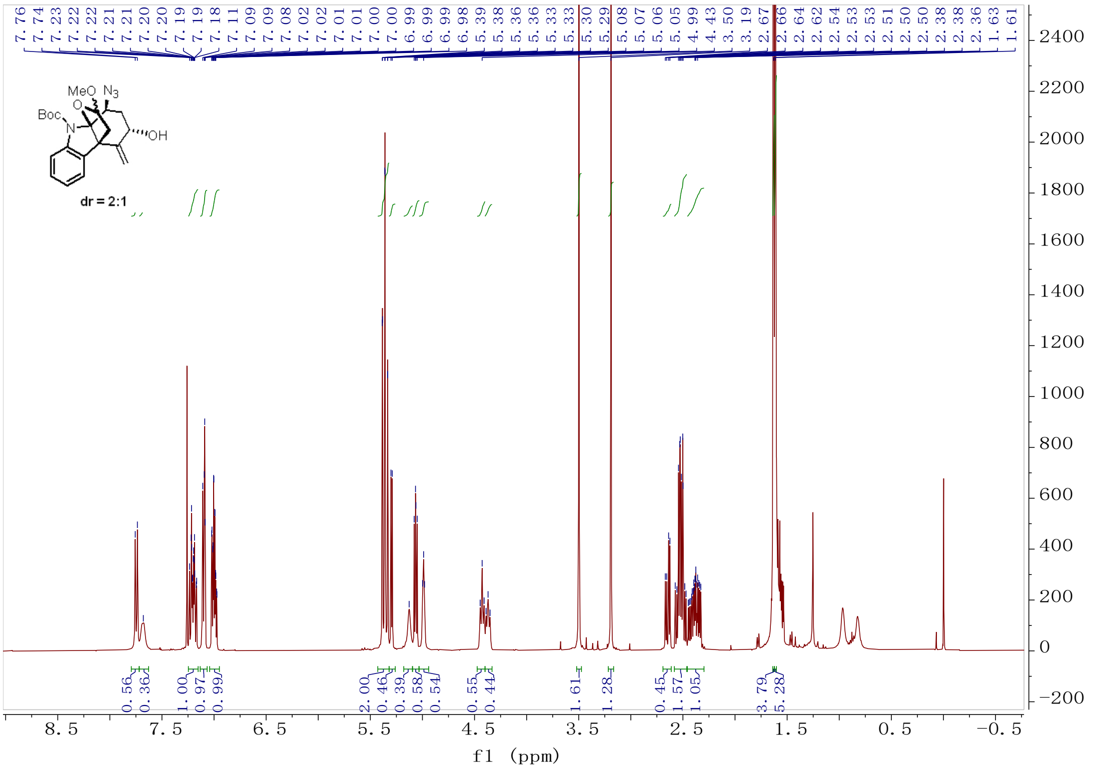

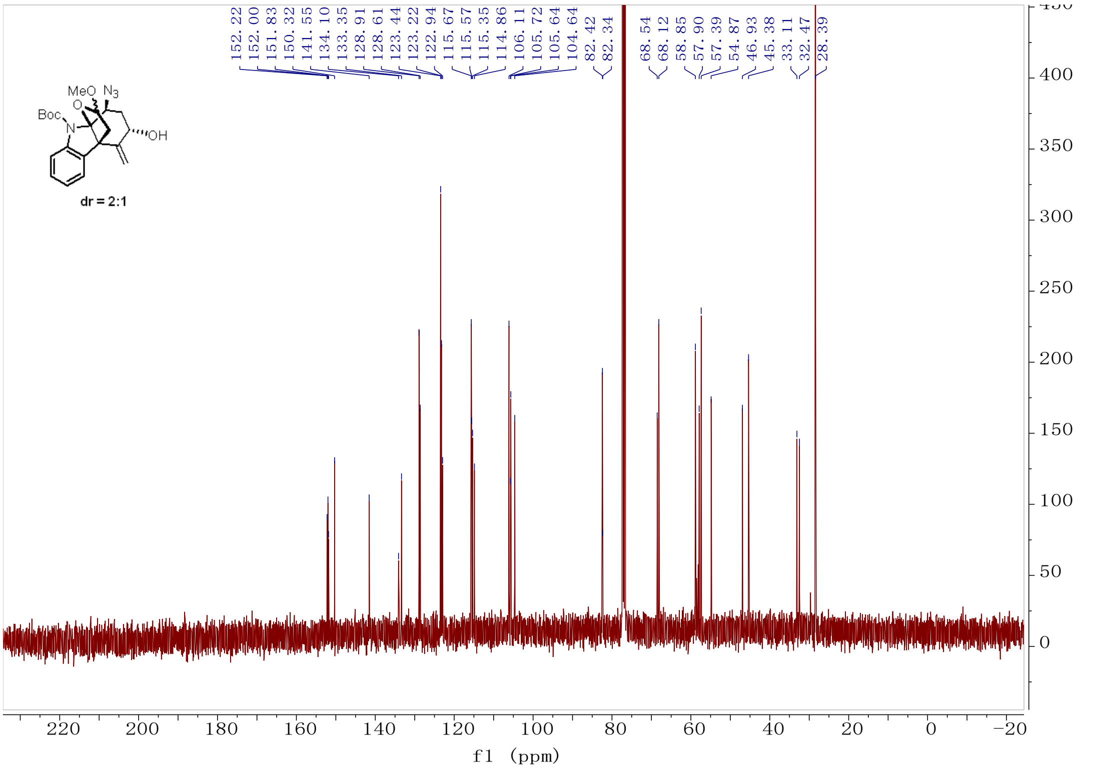

Compound **6**

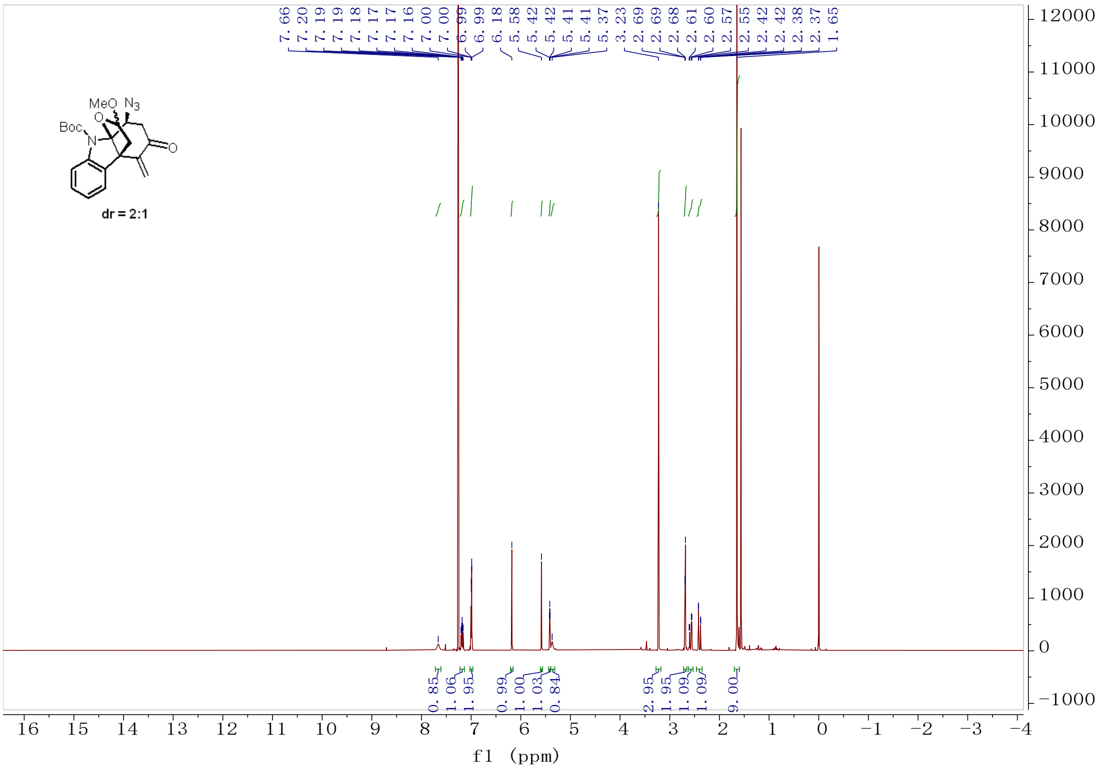
7.66 7.20 7.19 7.19 7.18 7.17 7.17 7.16 7.00 7.00 6.99 6.99 6.18 5.58 5.42 5.42 5.41 5.41 5.37 3.23 2.69 2.69 2.68 2.61 2.60 2.57 2.55 2.42 2.42 2.38 2.37 1.65
MeO N3
Boc
N
O
O
dr = 2:1
0.85 1.06 1.95 0.99 1.00 1.03 0.84 2.95 1.95 1.09 1.09 9.00
f1 (ppm)

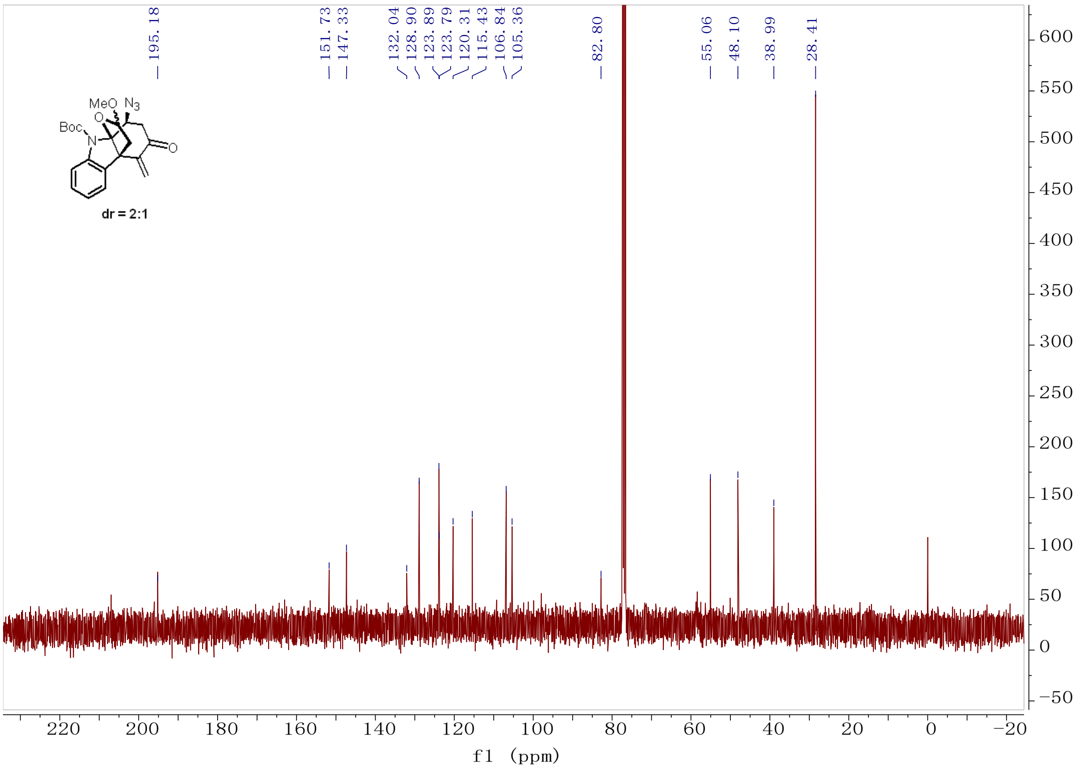
195.18
151.73 147.33
132.04 128.90 123.89 123.79 120.31 115.43 106.84 105.36
82.80
55.06 48.10 38.99 28.41
MeO N3
Boc
N
O
O
dr = 2:1
f1 (ppm)

Compound **8** (up)

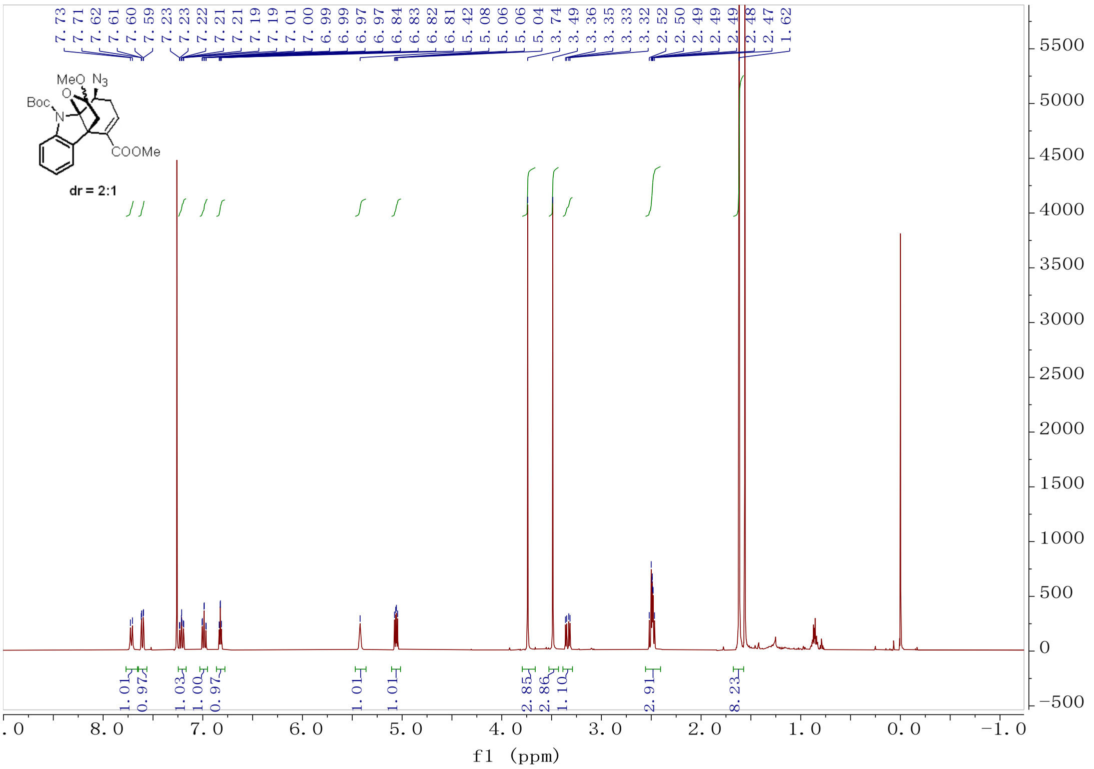

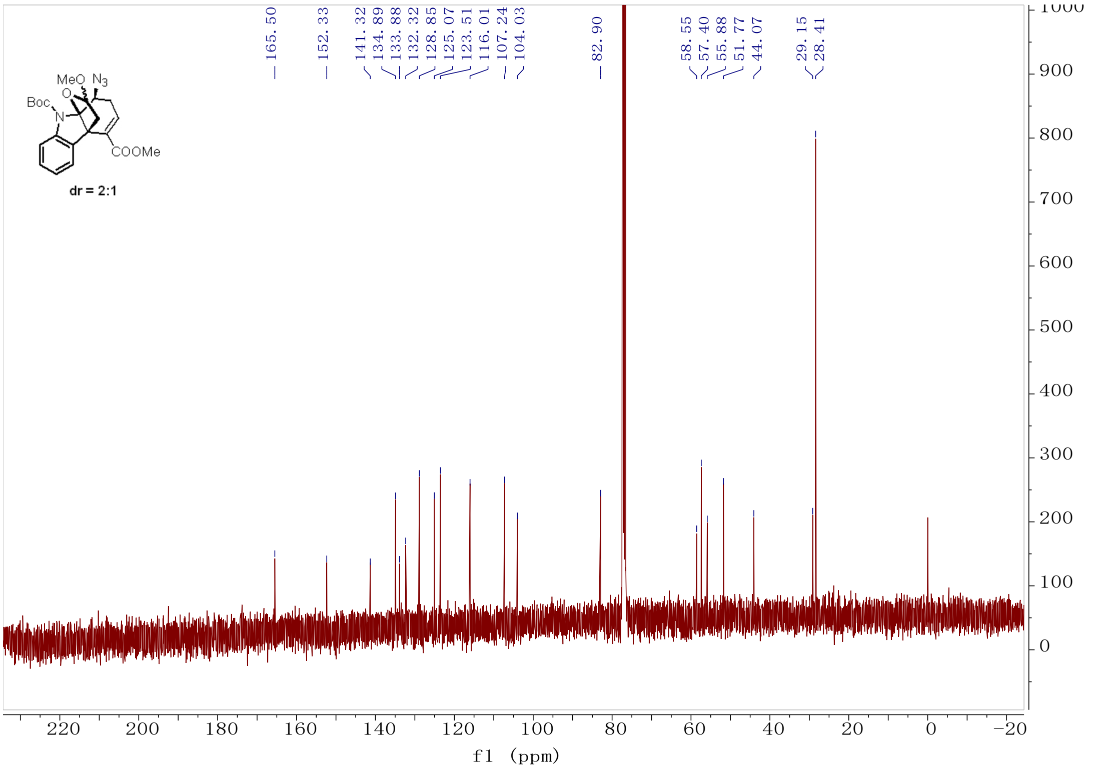

Compound **8**(down)

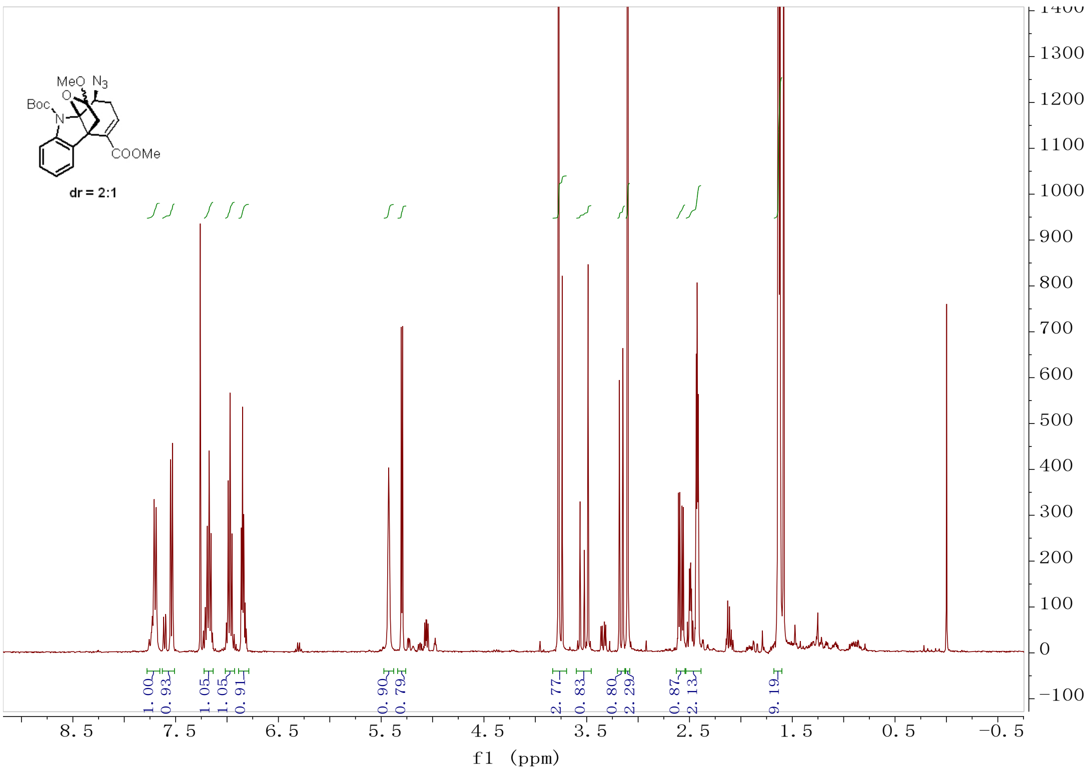

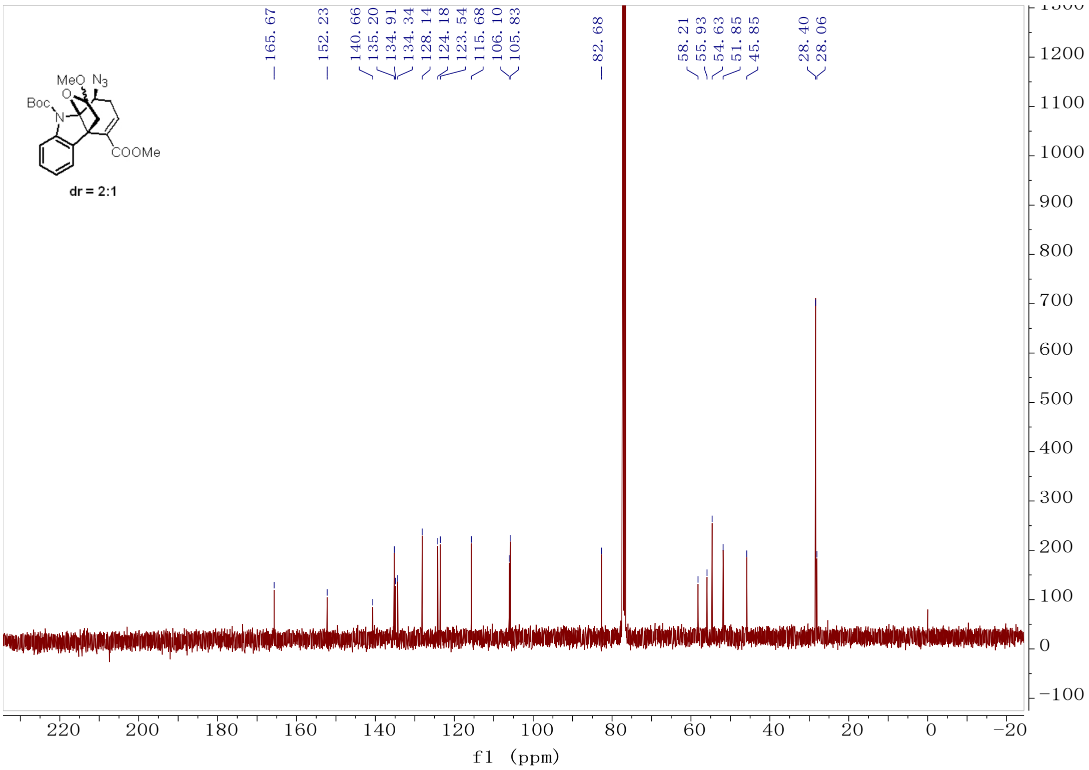

Compound **17a**

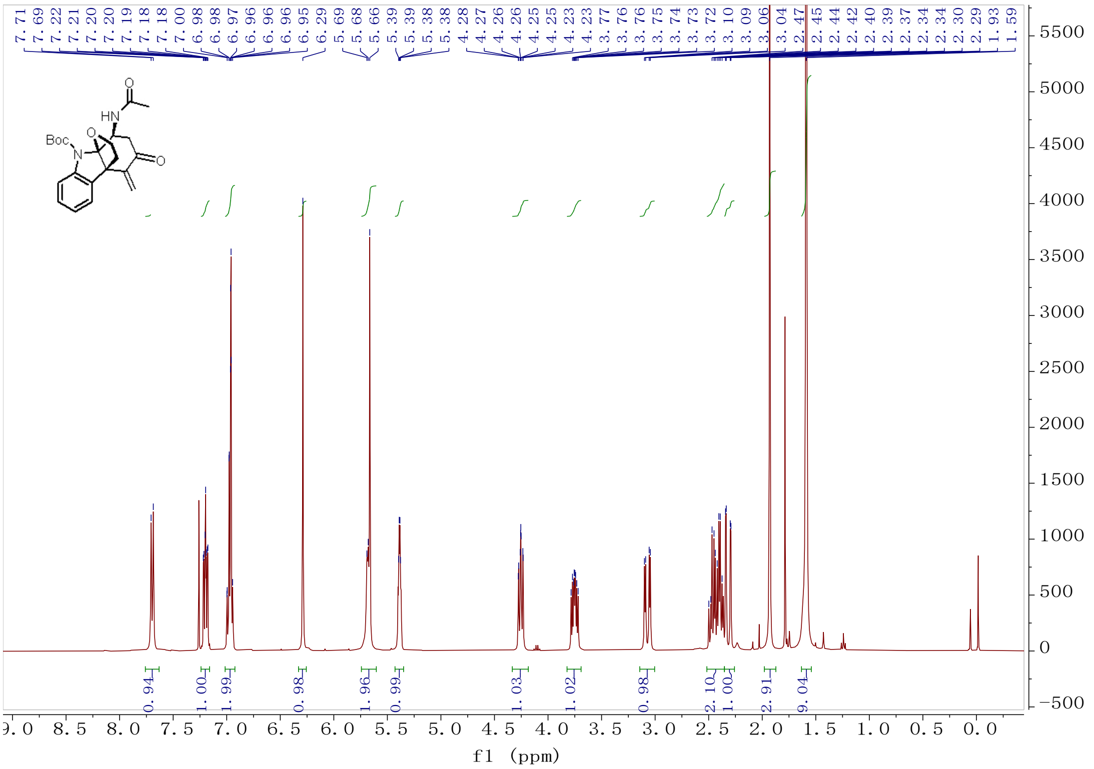

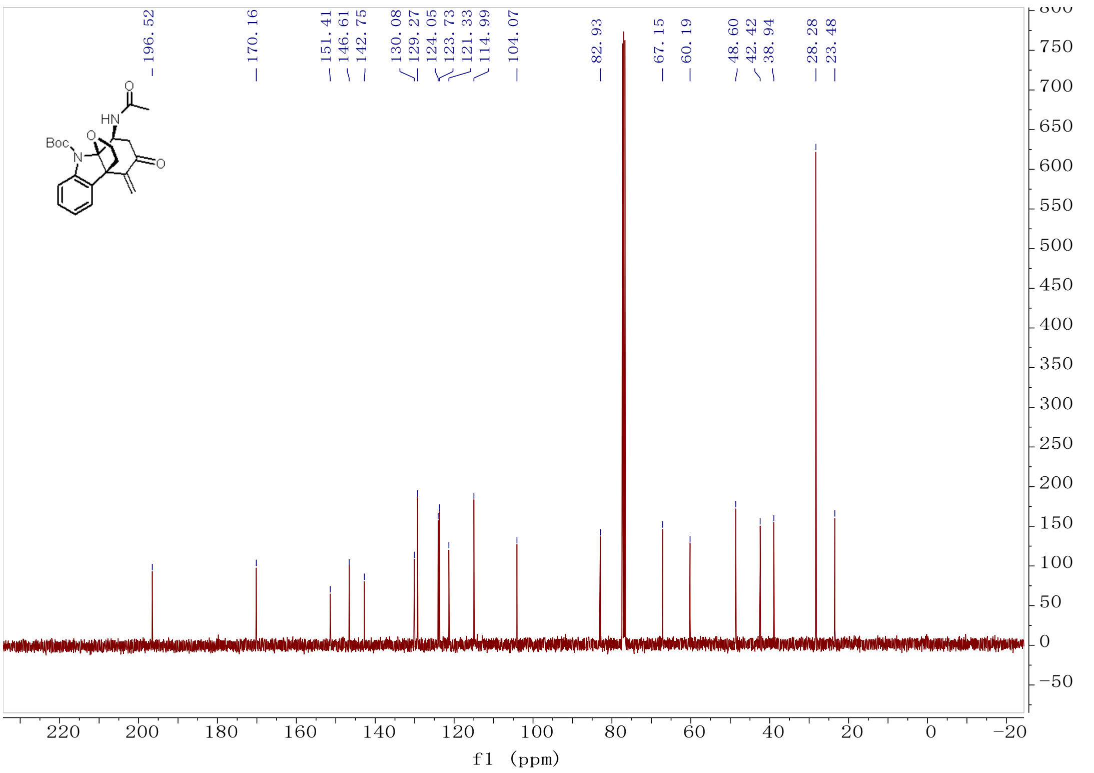

Compound **17b**

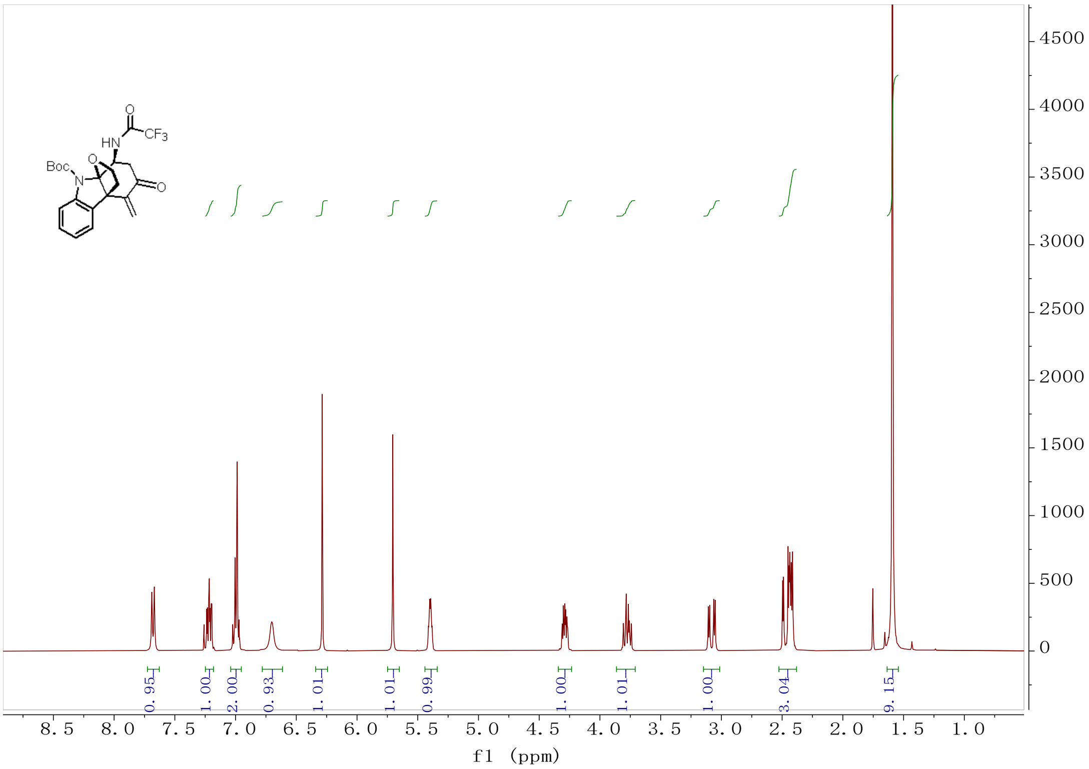

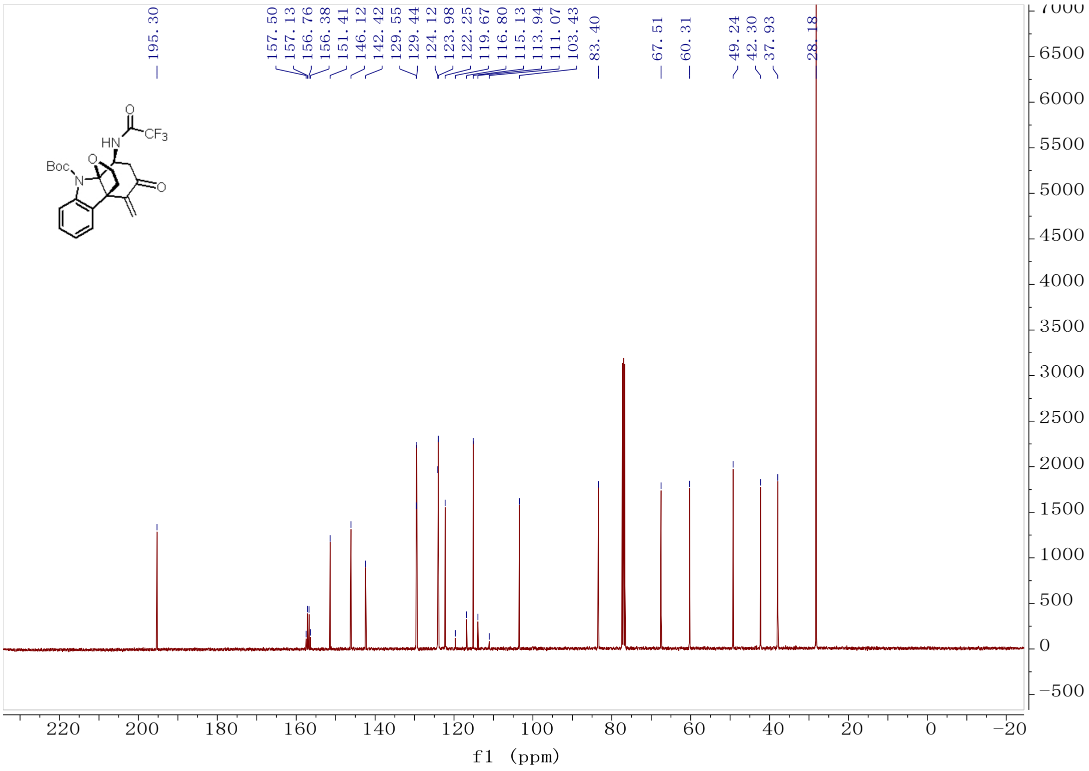

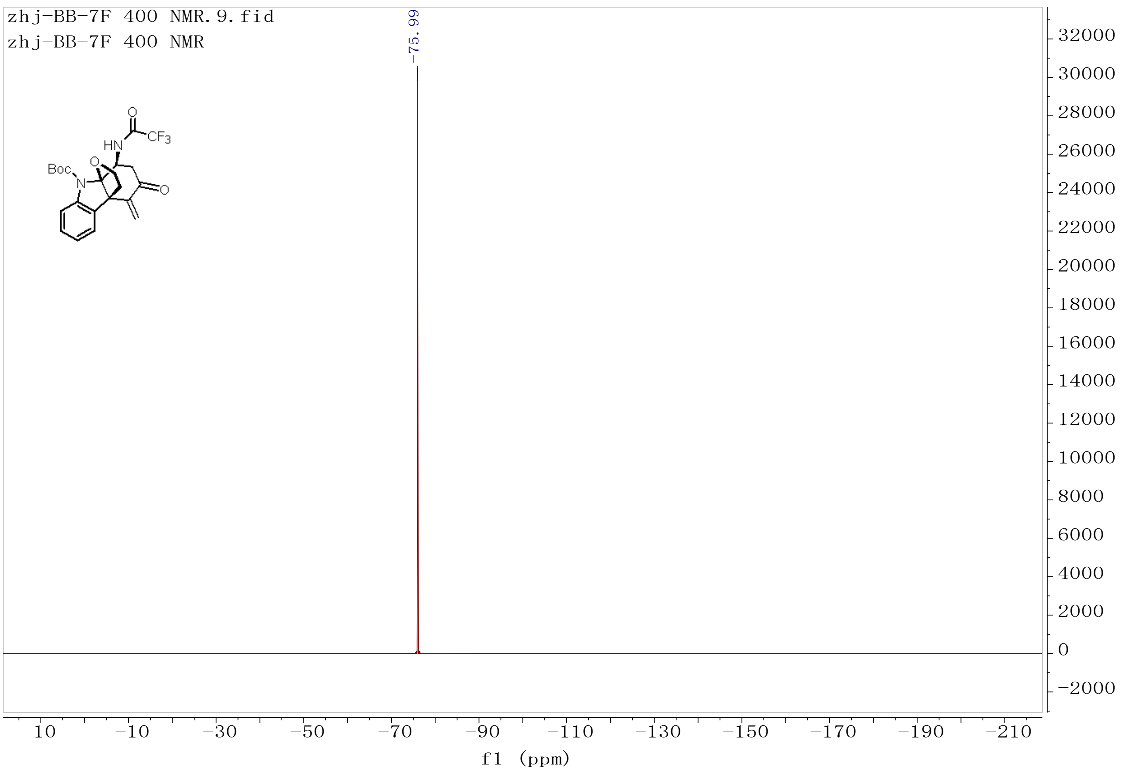

zhj-BB-7F 400 NMR.9.fid
zhj-BB-7F 400 NMR
−75.99
HN
CF3
Boc
N
O
O
32000
30000
28000
26000
24000
22000
20000
18000
16000
14000
12000
10000
8000
6000
4000
2000
0
−2000
10
−10
−30
−50
−70
−90
−110
−130
−150
−170
−190
−210
f1 (ppm)

Compound **17c**

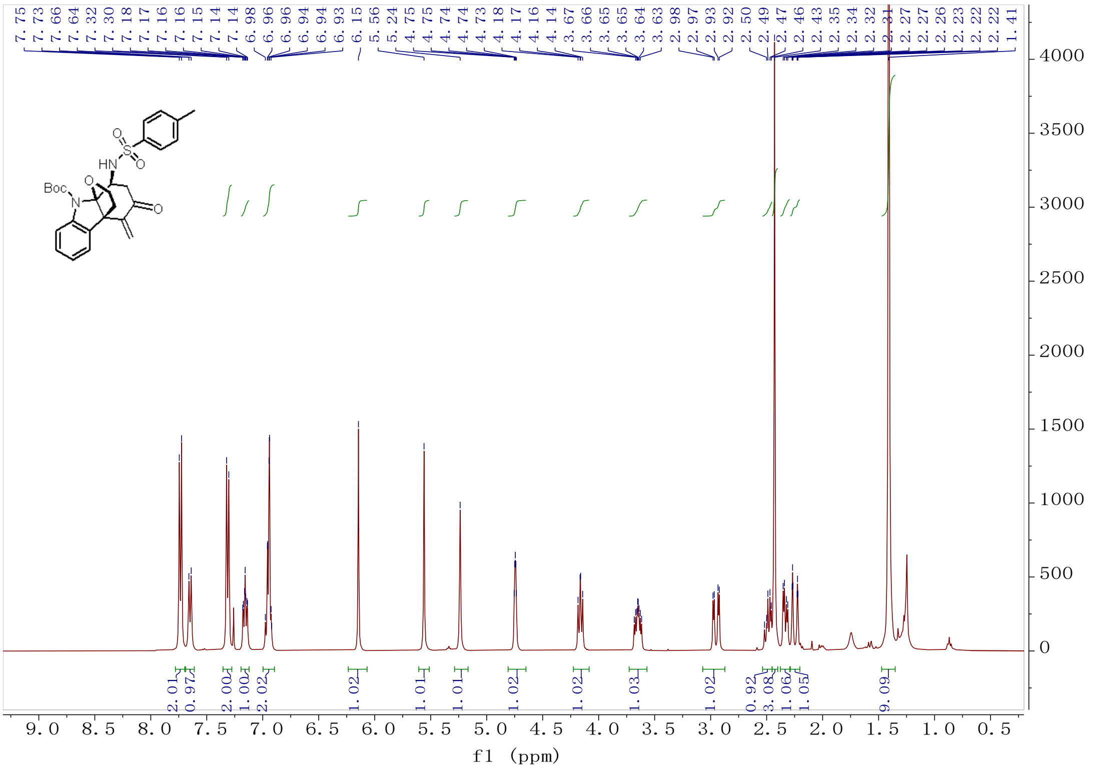

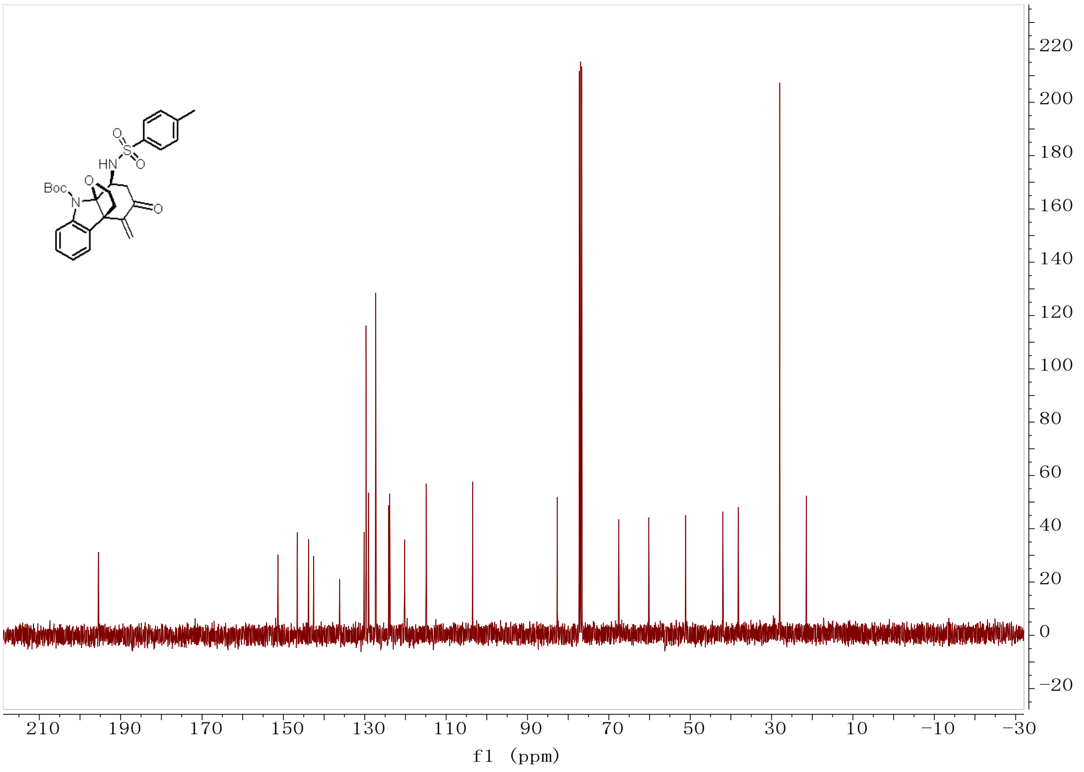

Compound **17d**

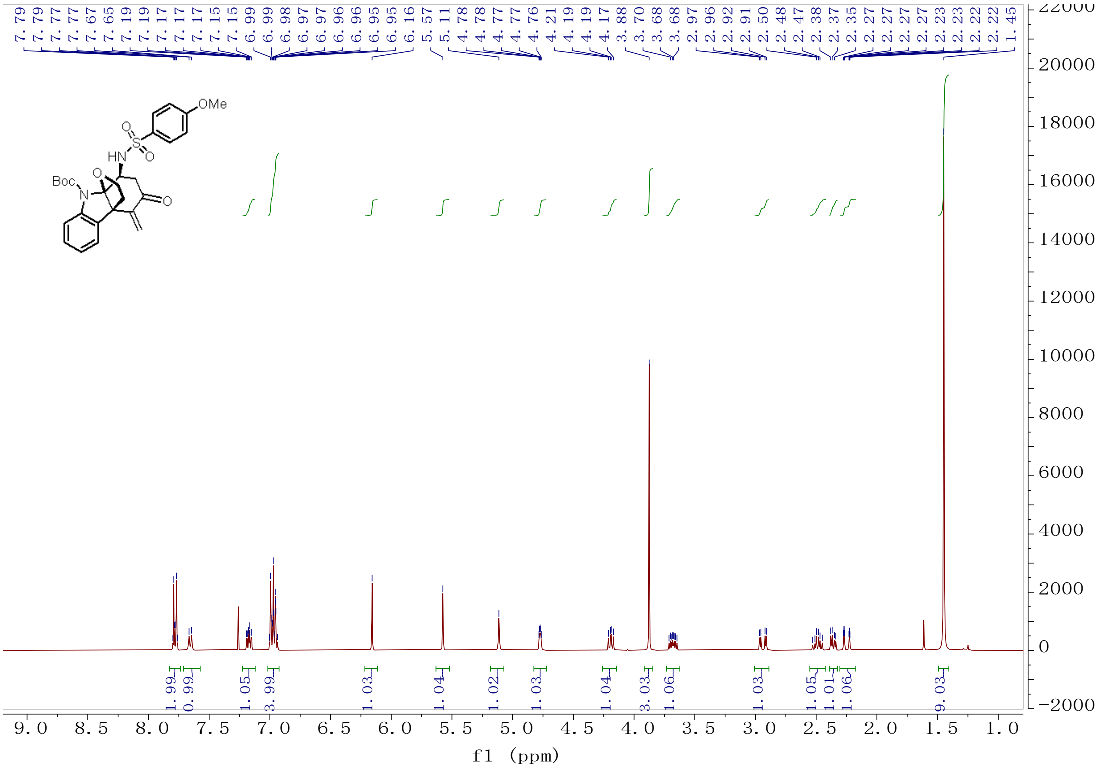

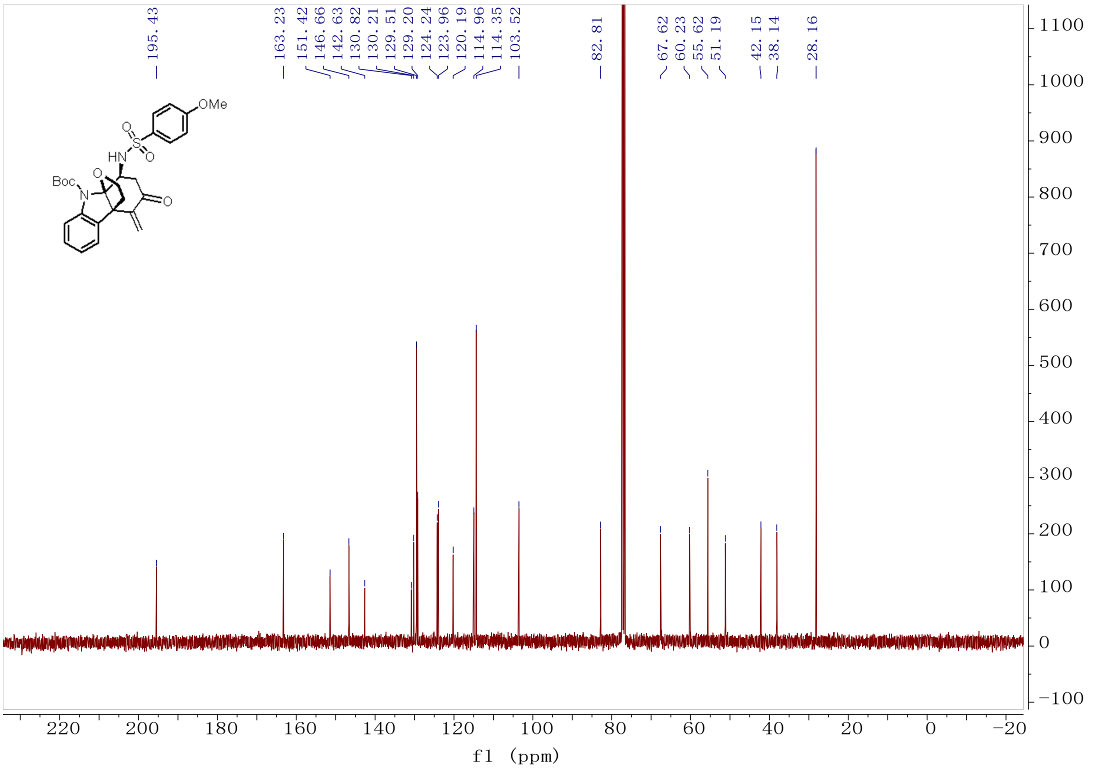

Compound **17e**

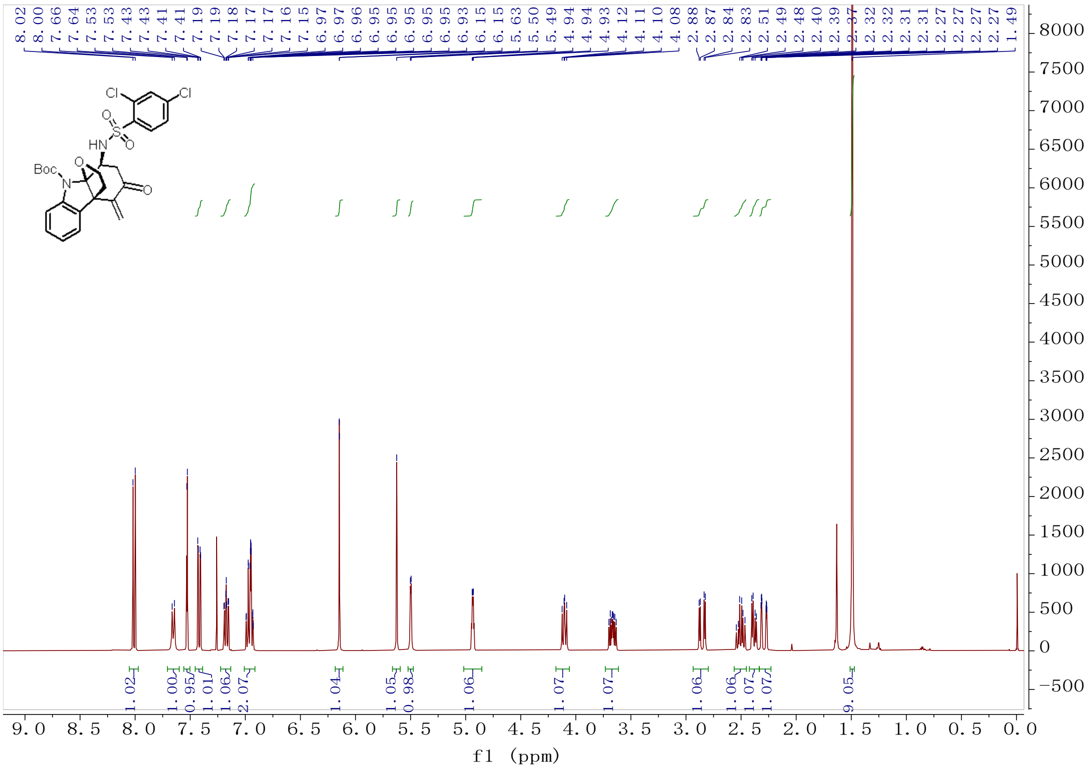

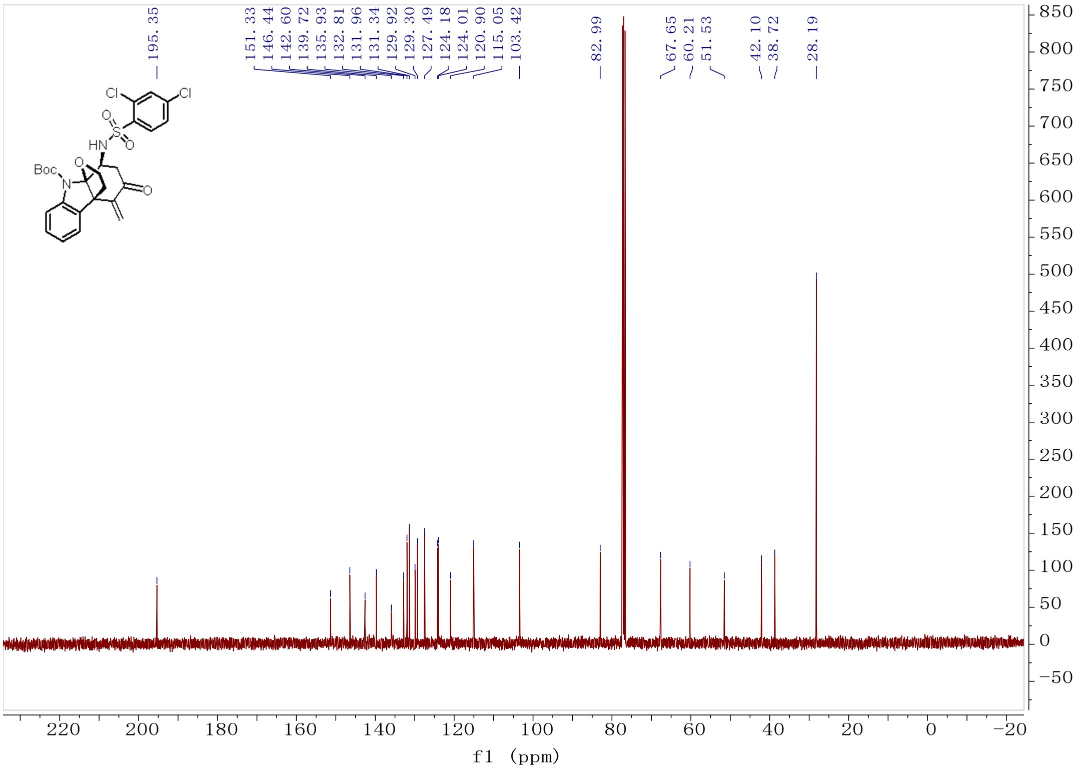

Compound **17f**

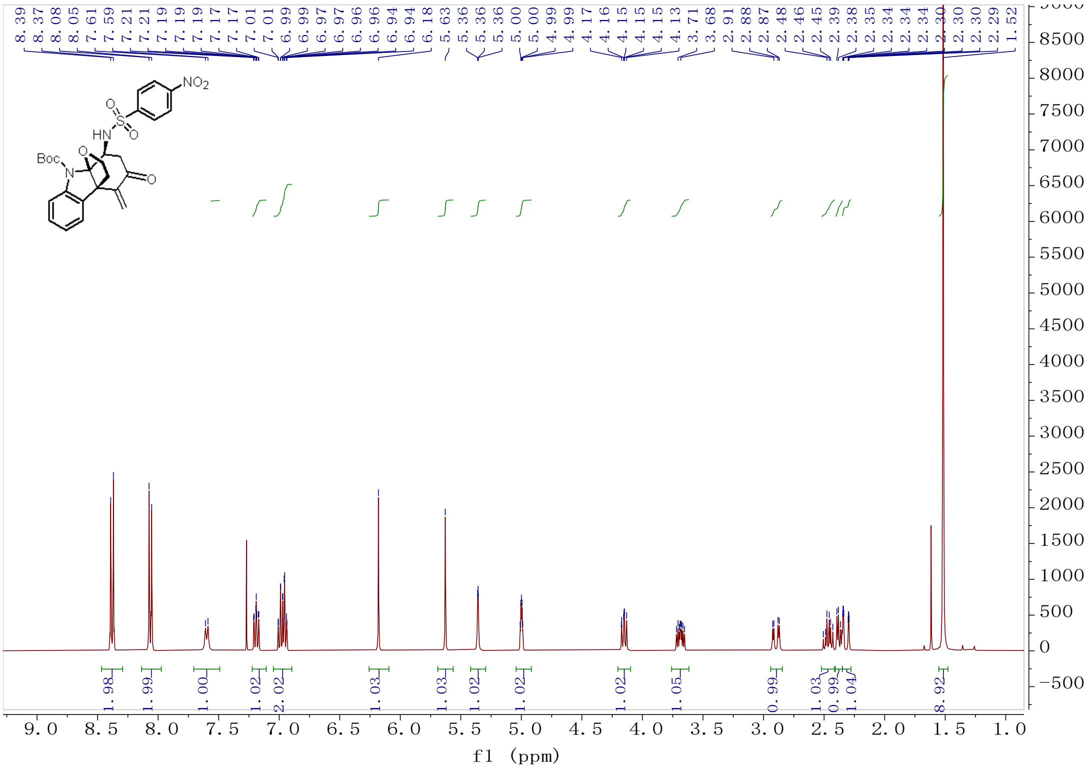

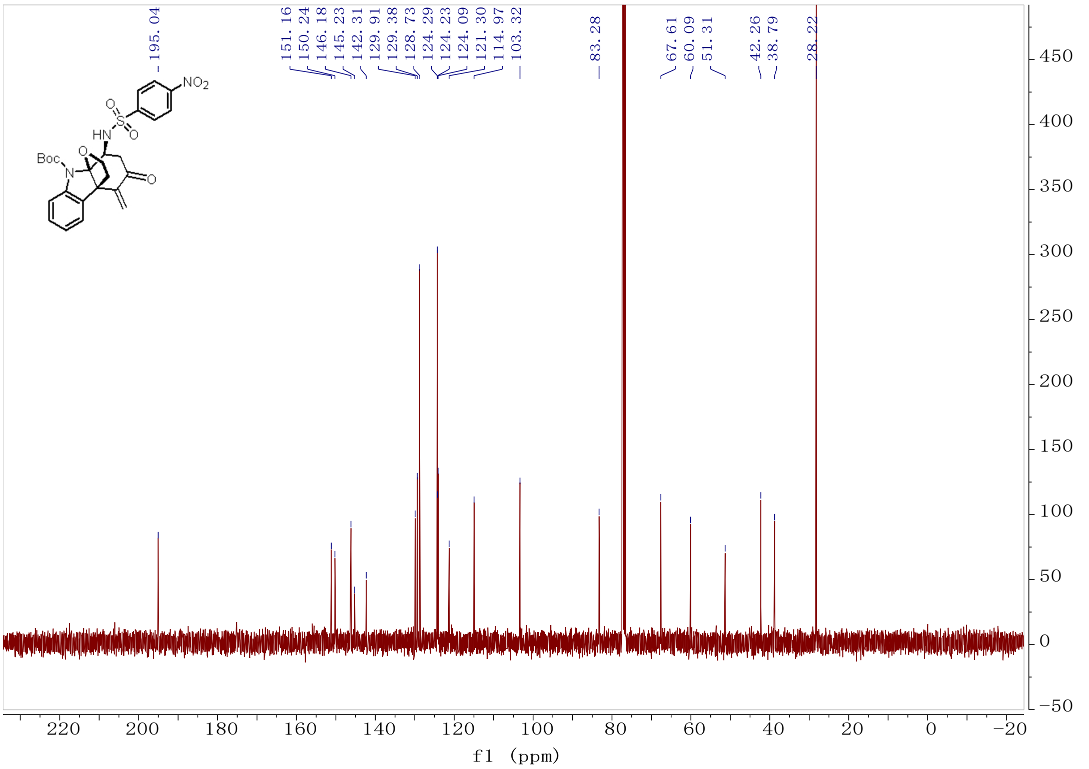

Compound **20a**

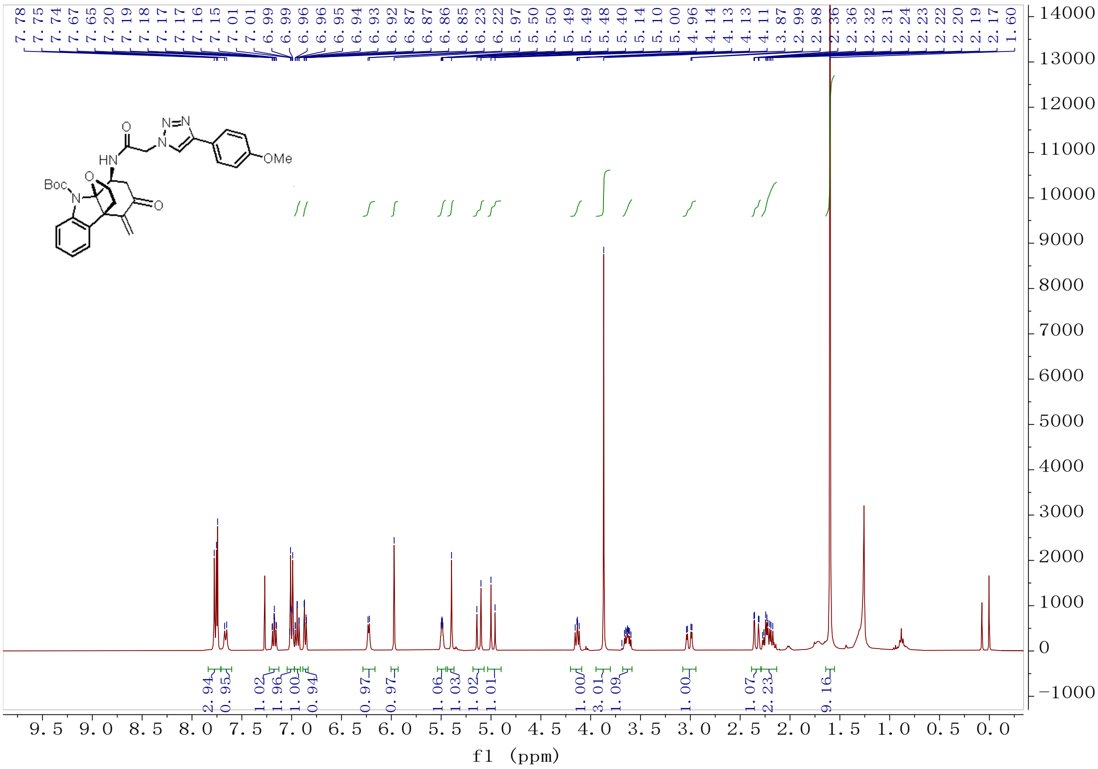

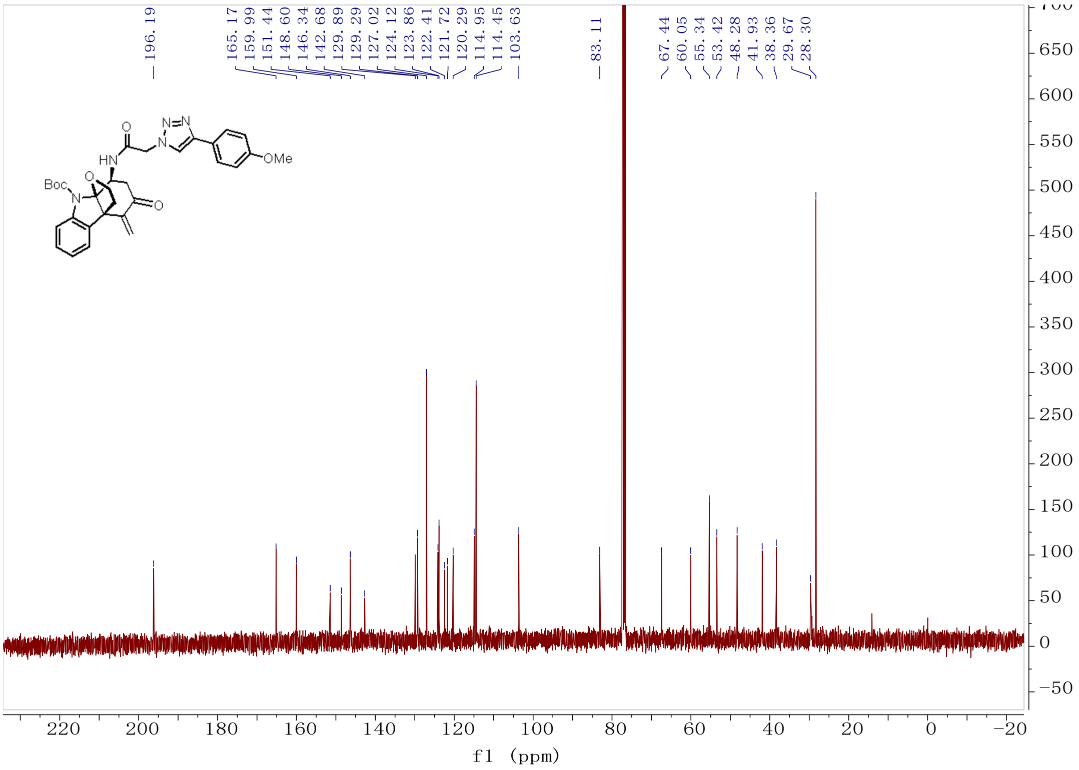

Compound **20b**

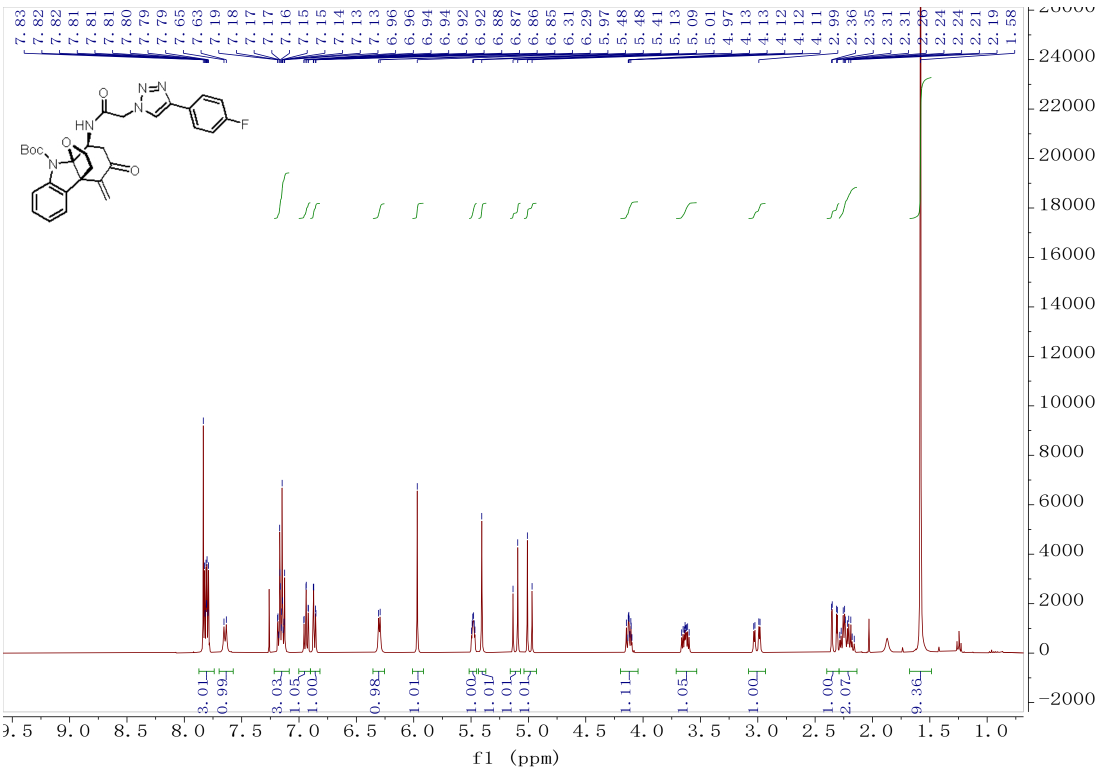

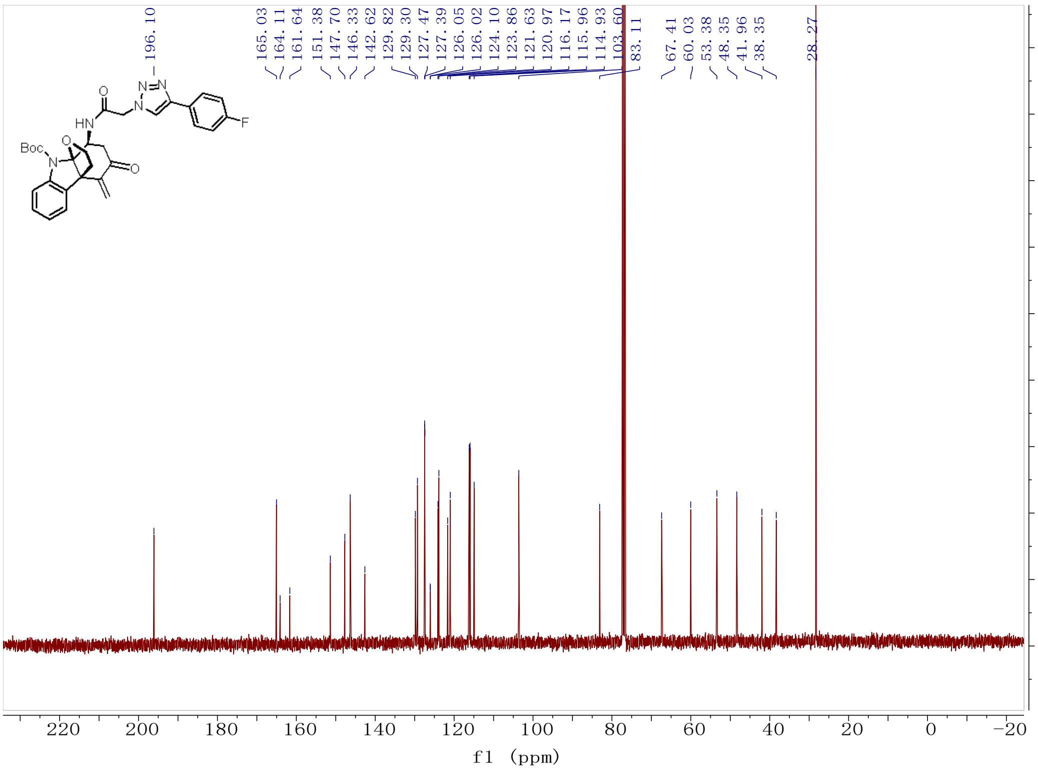

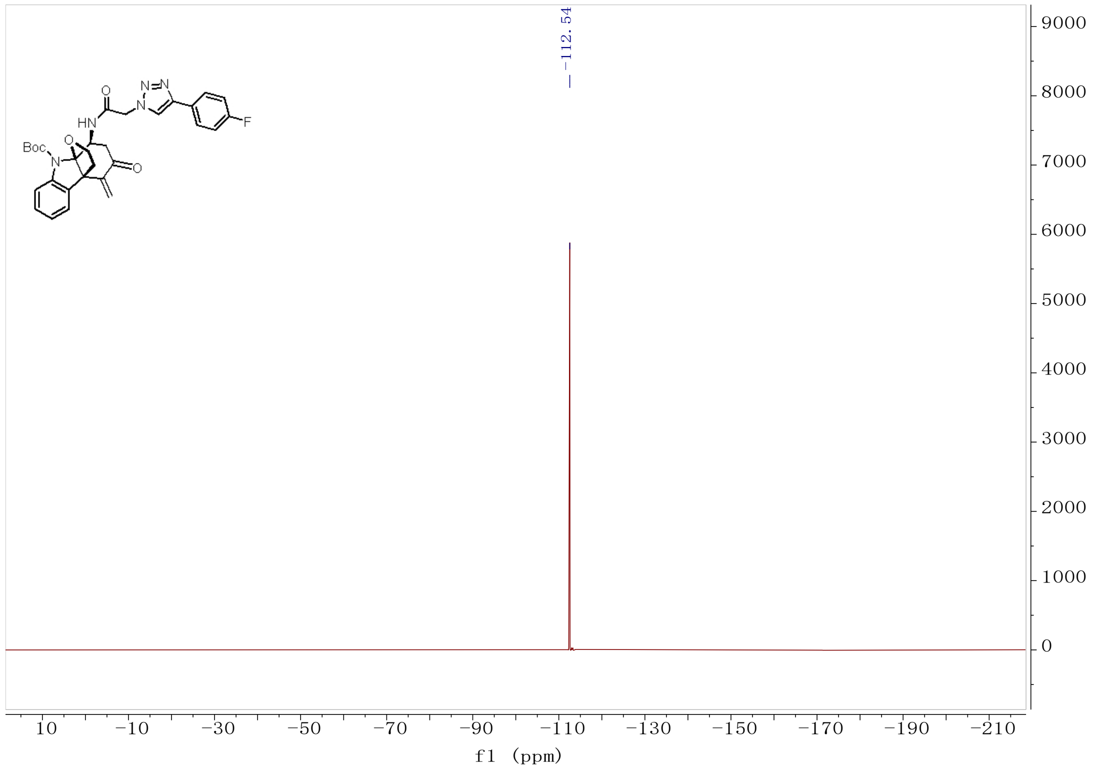

−112.54
Boc
N
O
HN
O
N
N=N
F
O
9000
8000
7000
6000
5000
4000
3000
2000
1000
0
10
−10
−30
−50
−70
−90
−110
−130
−150
−170
−190
−210
f1 (ppm)

Compound **20c**

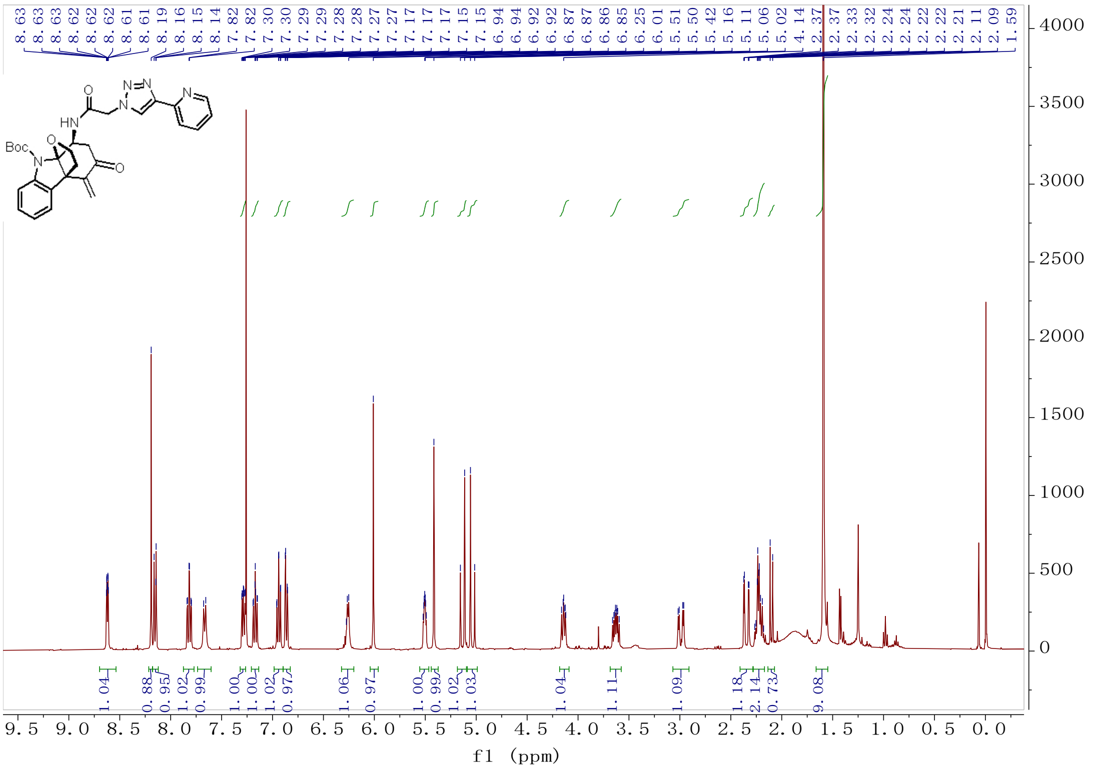

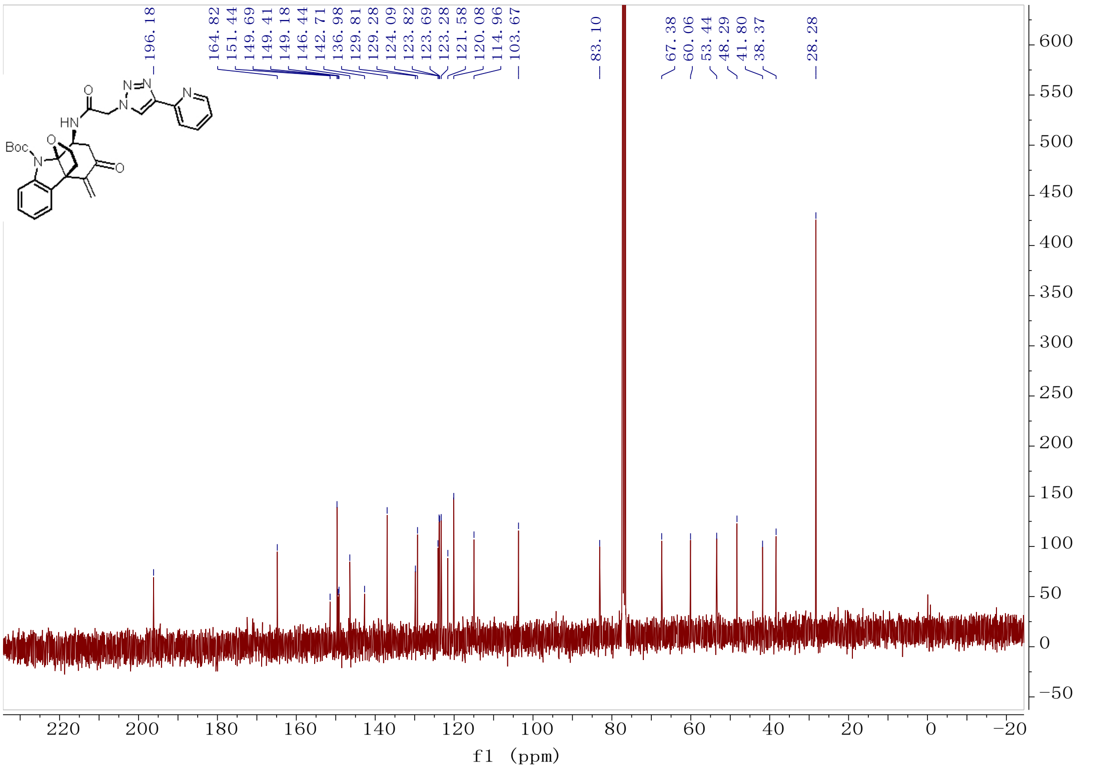

4. Details of Biological evaluation

MH7A cells were cultured in 10 cm^2 cell culture dishes with DMEM medium (Gibco, USA) containing 10% fetal bovine serum (Gibco, USA) at 37 °C in an incubator with CO_2 (5%). For cell viability detection, MH7A cells were collected and seeded in 96-well plates (5,000 cells per well) overnight. Then the adherent cells were stimulated with or without different dosages (2.5, 5, 10, and 20 μM) of compounds for 24 h. After treatment, cells were incubated with CCK-8 solution (10 μL/well) for 1 h. Finally, the absorbance of each well was tested through a microplate reader (450 nm; BioTek, USA). The viability rate of the control group was taken as 100%, and the 24 h-IC_{50} of each compound in MH7A was calculated according to the viability rate.

5. Reference

1. T. Wang, X. Duan, H. Zhao, S. Zhai, C. Tao, H. Wang, Y. Li, B. Cheng and H. Zhai, *Org Lett*, 2017, **19**, 1650-1653.
2. P. Zou, H. Yang, J. Wei, T. Wang and H. Zhai, *Org Lett*, 2021, **23**, 6836-6840.
