# Supplementary material for: Akuammiline alkaloid derivatives: divergent synthesis and effect on the proliferation of rheumatoid arthritis fibroblast-like synoviocytes
Source: Front Chem. 2023 Apr 28;11:1179948. doi: 10.3389/fchem.2023.1179948 (PMC10176115; doi:10.3389/fchem.2023.1179948)

Akuammiline alkaloid derivatives: divergent synthesis and effect on the proliferation of rheumatoid arthritis fibroblast-like synoviocytes

Xinye Bao, Jian Wei, Cheng Tao, Muhammad Adnan Bashir, Haijun Zhang, Bian Bao, Jian Chen, and Hongbin Zhai

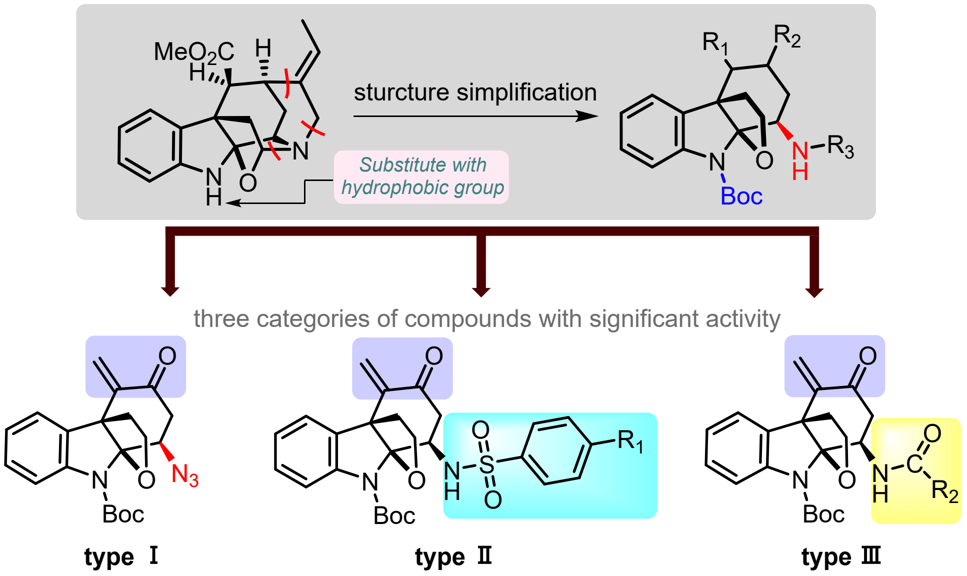

Supplement: Supplementary file 2 [file Image1.pdf]
